# A Carbene‐Stabilized Boryl‐Phosphinidene

**DOI:** 10.1002/chem.202200913

**Published:** 2022-04-13

**Authors:** Samir Kumar Sarkar, Subrata Kundu, Mohd Nazish, Johannes Kretsch, Regine Herbst‐Irmer, Dietmar Stalke, Parameswaran Parvathy, Pattiyil Parameswaran, Herbert W. Roesky

**Affiliations:** ^1^ Institut für Anorganische Chemie Universität Göttingen Tammannstrasse 4 37077 Göttingen Germany; ^2^ Department of Chemistry Indian Institute of Technology Delhi Hauz Khas New Delhi 110016 India; ^3^ Department of Chemistry National Institute of Technology Calicut Kozhikode Kerala 673601 India

**Keywords:** cyclic(alkyl)(amino)carbenes, bent geometry, boryl phosphinidenes, molecular orbitals, phosphaalkenes

## Abstract

Herein, the synthesis and characterization of the carbene‐stabilized boryl phosphinidenes **1**–**3** are reported. Compounds **1**–**3** are obtained by reacting Me‐cAAC=PK (Me_2_‐cAAC=dimethyl cyclic(alkyl)(amino)carbene) and dihaloaryl borane in toluene. All three compounds were characterized by X‐ray crystallography. Quantum mechanical studies indicated that these compounds have two lone pairs on the P center viz., an σ‐type lone pair and a “hidden” π‐type lone pair. Hence, these compounds can act as double Lewis bases, and the basicity of the π‐type lone pair is higher than the σ‐type lone pair.

## Graphical abstract

**What hides within**: Three different singlet boryl phosphinidenes were synthesized by using cAAC carbene, and their inversely polarized phosphaalkene characteristics were investigated. Quantum mechanical studies suggest that the P center of these boryl phosphinidenes contains two lone pairs viz., an σ‐type lone pair and a “hidden” π‐type lone pair.

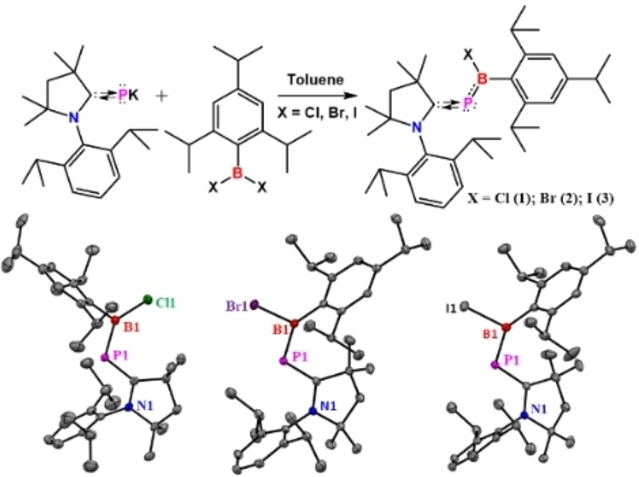

## Introduction

**Scheme 1:**
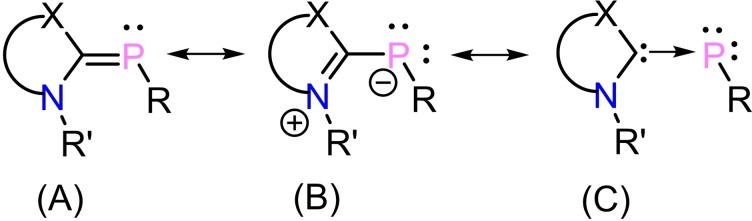
Canonical forms of carbene–phosphinidene adducts.

**Scheme 2:**
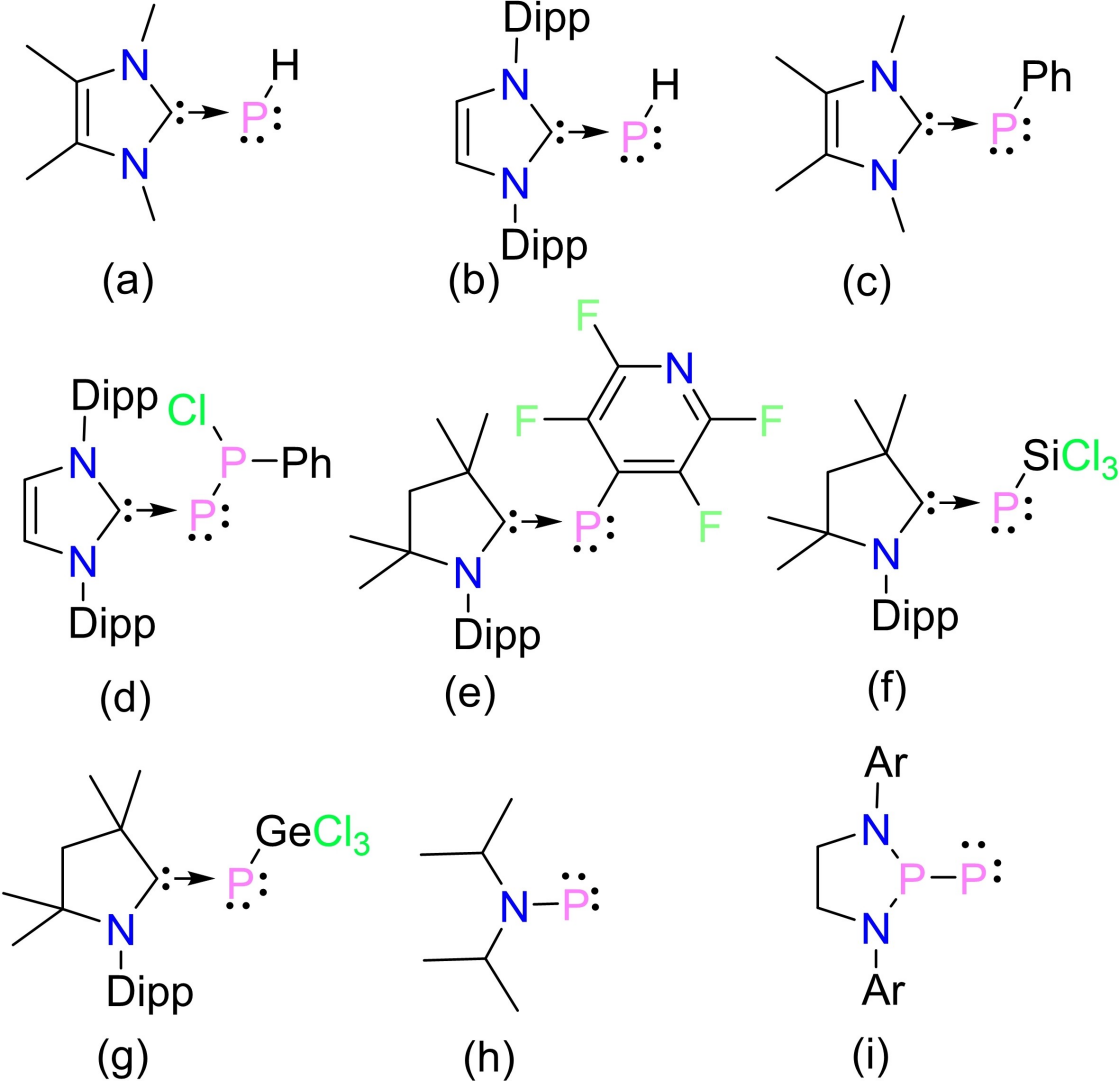
Examples of phosphinidenes (Ar=2,6‐bis[4‐*tert*‐butylphenyl)methyl]‐4‐methylphenyl).[^4^, ^5^]

Phosphinidenes (R−P̈:) are low‐valent phosphorus compounds analogous to carbenes and nitrenes, having a singly coordinated phosphorus atom containing six electrons in its valence level.^[1]^ Due to high reactivity, phosphinidene is a short‐lived species. Therefore, several strategies have been employed to stabilize phosphinidenes, including complexation with transition metals, substituting with π‐donating and sterically bulky groups, etc.[^1b^, ^1c^, ^1d^, ^1e^, ^1f^] Lappert et al., isolated the first structurally characterized di‐coordinated terminal transition‐metal‐complexed phosphinidene [R−P̈:→M(*η*‐C_6_H_5_)_2_] (M=Mo, W, R=2,4,6‐*t*Bu_3_‐C_6_H_2_).[^2a^, ^2b^] Several terminal phosphinidene complexes were reported with transition‐metal and substituents R on the P atom.^[2]^ Subsequently, metal‐free phosphinidenes (R−P̈:) were stabilized by Schmidpeter and Arduengo.[^3a^, ^3b^] In 1997, Arduengo et al. reported the N‐heterocyclic carbene supported phosphinidene [(NHC)P−Ph, NHC=1,3,4,5‐tetramethylimidazol‐2‐ylidene]. It was termed as an inversely polarized phosphaalkene and described by three canonical forms **A**–**C** (Scheme 1).^[3]^ Form **A** contains a formal P=C double bond, **B** is a zwitterionic form with a P−C single bond and **C** shows a dative C→P bond with two lone pairs of electrons on the P atom. Numerous carbene (NHC/cAAC) stabilized phosphinidenes (R−P̈:) have been reported, and their structure, electronic properties and reactivity vary with substituent R (Scheme 2, **a**‐**g**).[^4^, ^5^]

**Scheme 3:**
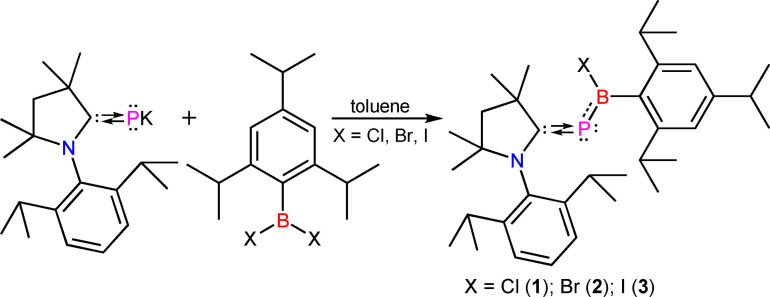
Synthesis and chemical structures of **1**, **2**, and **3**.

In 2012, Cummins et al. reported a mono‐coordinated phosphinidene (*i*Pr_2_NP̈:), which was formed as a transient intermediate while thermolysis of dibenzo‐7‐phosphanorbornadiene (Scheme 2, **h**).^[1f]^ Using the thermodynamic and kinetic stability of bulky ligand, Bertrand et al. synthesized a room temperature stable singlet phosphino‐phosphinidene compound (Scheme 2, **i**). The reactivities of phosphino‐phosphinidene were studied using several nucleophiles such as CO, isocyanides, carbenes, and phosphines, etc. –^[1g–f]^ Recently, Chen et al. reported the synthesis and structure of a terminal scandium boryl phosphinidene complex which showed strong Sc−P π bonding.^[2l]^ The influence of substitution and the coordination of phosphinidene to carbene/metal play a significant role in bonding and chemical properties. Therefore, the isolation of new phosphinidene derivatives is an exciting area of research. In this communication, we report the synthesis of the carbene stabilized boryl phosphinidenes (Scheme 3, **1**–**3**) which are also characterized as inversely polarized phosphaalkenes. DFT calculations are employed to obtain further insight into the bonding and structure–property relationship of the newly developed carbene‐stabilized boryl phosphinidenes.

## Results and Discussion

Compounds **1**–**3** were accessed from the reaction between Me‐cAAC=PK^[6]^ and di‐haloaryl borane (Trip‐BX_2_, X=Cl, Br, I) in a 1 : 1 molecular ratio.

The block‐shaped yellow crystals of **1**, **2**, and **3** were obtained in good yield at 0 °C from the concentrated toluene solution. Compounds **1**–**3** were fully characterized by NMR (^1^H, ^13^C, ^11^B, and ^31^P) and mass spectrometry (LIFDI). All these compounds are highly stable in the solid‐state and in solutions at room temperature under an inert atmosphere for months. However, they are unstable under ambient conditions and slowly change their intense yellow color to a colorless form. Single‐crystal X‐ray diffraction and elemental analysis confirmed the compositions of **1**, **2**, and **3**.

^13^C, ^31^P, and ^11^B NMR spectroscopy provided a wealth of structural information on the inversely polarized phosphaalkene. The ^13^C{^1^H} signal of C=P unit of inversely polarized phosphaalkenes appeared at 220.9 (d, *J*_c‐p_=91.9 Hz), 220.8 (d, *J*_c‐p_=94.5 Hz), and 220.7 (d, *J*_c‐p_=104.6 Hz) ppm for compounds **1**, **2**, and **3** respectively. The observed ^13^C{^1^H} NMR signals and *J*_C‐P_ coupling constants for the C=P units are similar to the reported phosphaalkenes.^[6b]^ The ^31^P NMR shifts for the compounds are strongly affected by the electronic nature of the substituents and the hybridization of the phosphorus atom. The introduction of an electropositive boryl group provides a deshielding effect on the phosphorus chemical shift. Also, the halogen atoms such as Cl, Br, and I on the boron center typically move the chemical shift significantly downfield. The ^31^P{^1^H} NMR signal appeared at 35.5, 47.1, and 66.9 ppm for compounds **1**, **2**, and **3**, respectively. This observation is in accordance with the increasing order of Lewis acidity of the boron center, which increases when the halogen changes from Cl to I.^[6c]^ Thus, the ^31^P{^1^H} NMR spectroscopy supports the formation of delocalized C−P−B π‐system and the extent of delocalization increases from compound **1** to **3**. Note that the ^31^P chemical shift values move downfield compared to inversely polarized phosphaalkenes.^[6d]^ For instance, the ^31^P chemical shifts for phosphatriafulvenes show inverse polarity around −23.2 to −74.1 ppm.^[6]^ The quadrupolar characteristics of the ^11^B{^1^H} nucleus and the asymmetric local fields in compounds usually result in broad ^11^B{^1^H} NMR resonances. The ^11^B{^1^H} NMR resonances appear at 72.83, 71.81, and 69.80 ppm for the compounds **1**, **2**, and **3**, respectively. The ^31^P and ^11^B NMR data indicate that the B−P bond has considerable double‐bond character and ^31^P and ^11^B resonances are similar to those reported phosphinidene boranes (R−B=P−R).^[6]^

**Figure 1:**
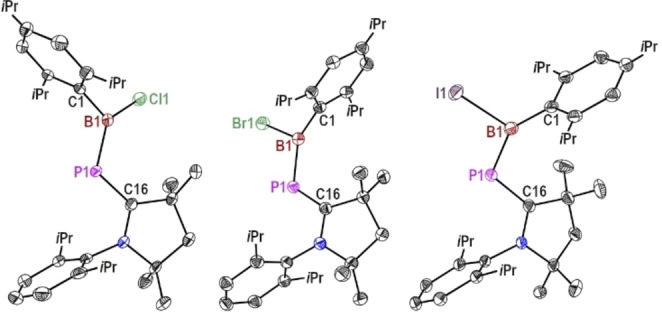
Molecular structures of (left to right) **1**, **2**, and **3**; hydrogen atoms and alkyl substituents are omitted for clarity. Anisotropic displacement parameters are depicted at the 50 % probability level.

The solid‐state structures of complexes **1**, **2**, and **3** consist of a C−P−B backbone with an almost coplanar halogen (X=Cl, Br, I) atom (Figure 1). The bond parameters are listed in Table 1. The observed dihedral angles of C_carbene_−P−B−X are 18.32 (17)° (**1**), 179.02 (6)° (**2**), and 177.70 (6)° (**3**). The C=P=B skeleton is significantly bent with a bond angle of 114.68°, 111.94°, 110.78° and the dihedral angles between C17−C16−N1 and X1−B1−C1 planes are 25.06°, 21.43° and 31.50° for compounds **1**, **2**, and **3**, respectively.

**Table 1:** Experimental (calculated) structure parameters for compounds **1**–**3**.

Parameter	**1**	**2**	**3**
C−P bond length	1.7709(15) Å (1.768)	1.7850(14) Å (1.774)	1.7944(14) Å (1.779)
B−P bond length	1.8607(18) Å (1.873)	1.8607(15) Å (1.870)	1.8456(15) Å (1.860)
C−P−B bond angle	114.68°(14) Å (114.3°)	111.94°(14) Å (111.9)	110.78°(15) Å (111.5)

The molecular structures reveal that in the solid state, the B1−Cl1 (1.8046(18) Å) bond is *cis* to the C−P bond (C16−P1 1.7709 (15) Å) in complex **1**, whereas the B−X bonds in complexes **2** (B1−Br1 2.0085(15) Å), and **3** (B1−I1 2.2504 (15) Å) are in *trans* position to the C−P bonds, respectively. Furthermore, the nearly anti‐periplanar alignment represented by torsion angles N−C−P−B (**1** 172.74(12)°, **2** 164.82(9)°, **3** 157.78(10)°) decrease from species **1** to **3**. The P1−C16 (**1** 1.7709 (15) Å, **2** 1.7850 (14) Å, **3** 1.7944(14) Å) bond lengths are longer than reported double bond lengths in phosphaalkenes (1.61–1.71 Å)^[7a]^ but are shorter than P−C single bonds (1.85 Å),^[7b]^ thus indicating some degree of π‐bonding.

Similarly, P1−B1 (**1** 1.8607(18) Å, **2** 1.8607(15) Å, **3** 1.8456(15) Å) bond lengths are elongated compared to phosphaborenes (1.8211(16)–1.8309(16) Å)^[8a]^ or phosphinidene boranes (1.795(3)–1.8067(15) Å)^[8b]^ comprising of a P=B bond. On other hand, P−B bonds are shortened as compared to that in [(*tert*‐butyl‐1‐phosphaethene)(diethylhydroborato)titanocene] (2.054(3) Å)^[8c]^ and NHC‐stabilized phosphinidene bis‐BH_3_ complex (1.931(4)–1.943(4) Å)^[8d]^ that contain a P−B single bond and an adjacent P=C bond. Among the three compounds (**1**–**3**), the most elongated P−C bond length (1.7944(14) Å) and the shortest B−P bond length (1.8456(15) Å) were found in **3** (X=I). This is due to the high Lewis acidity of the boron center of compound **3** compared to **1**–**2** (X=Cl and Br), and the phosphinidene lone pair being relatively more engaged in donating to the boron center and compared to back‐bonding to the carbene center of cAAC. Additionally, C_carbene_−N bond lengths in **1**–**3** (**1** 1.3352(19) Å, **2** 1.3379(16) Å, **3** 1.3306(16) Å) are elongated (1.9–2.5 %) compared to that in free cAAC (1.3053(13) Å),^[8e]^ which points out that phosphorous is a better π‐donor than nitrogen atom in cAAC. Therefore, the NMR spectroscopy and crystal data analysis suggest the presence of inversely polarized phosphaalkene character.

Quantum mechanical calculations were carried out at the M06‐2X/def2‐TZVPP//BP86/def2‐TZVP level of theory by incorporating Grimme empirical dispersion correction with Becke–Johnson damping (GD3BJ) to explore the bonding and reactivity of the molecules **1**–**3**.^[9]^ The formation of **1**–**3** are exothermic and the energy increases on changing the halogen from Cl (−19.20 kcal mol^−1^) to I (−38.14 kcal mol^−1^). The isomer **1**′, where the B−Cl bond is oriented similar to B−X bonds in complexes **2** and **3**, is less stable by 1.82 kcal mol^−1^. It can be attributed to the shorter P−Cl distance (2.97 Å), resulting in strong repulsions between the lone pairs on P and Cl centers (Figure S20 in the Supporting Information).

**Figure 2:**
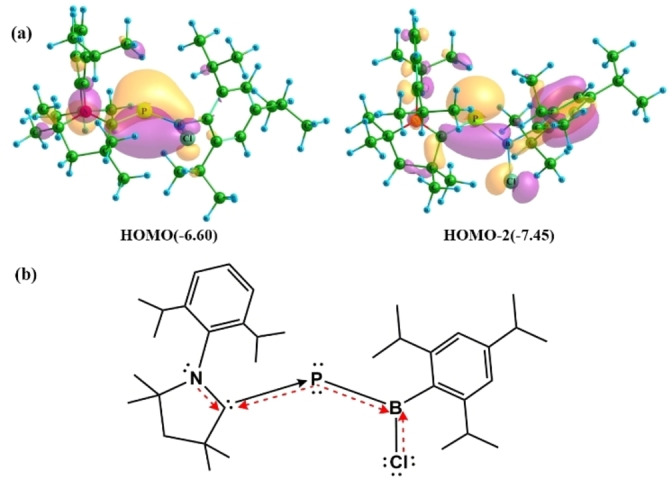
a) Important frontier molecular orbitals of **1**. Eigenvalues [eV] are given in parenthesis. The isosurface value is 0.03. b) Bonding representation in complexes **1**–**3**; Red bonds indicate the π‐framework in the N−C−P−B−X backbone, and black bonds indicate the σ‐framework. Straight lines indicate electron‐sharing bonds. Arrows indicate donor–acceptor bonds.

The molecular orbital (MO) analysis of **1** reveals that HOMO‐2 is a σ‐type lone pair orbital with a larger contribution from the P atom (Figure 2a). The NBO analysis shows a lone pair on the P atom with 54.2 % contribution from 3 s orbital and 45.6 % contribution from 3p orbitals. The lone pair with relatively less %p character can be correlated with a significantly bent C−P−B backbone. The extent of bending increases as the halogen is changed from Cl to I (see below). HOMO is a π‐type MO delocalized over the N−C−P−B−X backbone with a major coefficient from the P center. It can be formed by donating a lone pair of electrons from the P center to the antibonding C←N and B←X π*‐MOs. However, it is seen as a strong second‐order interaction from P−C π‐NBO to partially filled p‐type orbital on B in the NBO analysis. The bond occupancy from the NBO calculations indicates that the P‐atom contributes 66 % to the P=C π interaction. This suggests a polarized π bond with a larger contribution on the P atom. The MO and NBO analyses corroborated well with the very low Wiberg bond indices for the C_carbene_−P (1.28–1.35), P−B (1.31–1.41) and C_carbene_−N (1.29–1.32) bonds. Note that these Wiberg bond indices are lower than the C_carbene_−N Wiberg bond index of free cAAC (1.52; see below). The difference in electronegativity also induces polarity in the C_carbene_−P, P−B and C_carbene_−N bonds, reducing the bond index values. These bond index values support the partial double bond character observed in these bonds. The overall high positive group charge on cAAC (0.14–0.18) implies that σ‐donation from cAAC is higher than the π‐back donation from the P center. The NBO analysis shows considerable polarization of the P−C_cAAC_ σ‐MO and π‐MO in opposite directions (Table S8), thus indicating the inversely polarized phosphaalkene nature. However, the net polarization is minimal, as seen from the natural charges on C_cAAC_ (0.10–0.13 e) and P (0.06–0.08 e).

Three major interactions are responsible for the geometry of the compounds viz., the donation of σ‐lone pair on P to B center, the donation of lone pair of electrons from halogen atom to B center, and the donation from P−C π‐NBO to the B center. The strength of interaction corresponding to the donation of σ‐lone pair on P to B center is 5.4 kcal mol^−1^ when halogen atom is Cl (**1**), 2.6 kcal mol^−1^ for Br (**2**), and 2.7 kcal mol^−1^ for I (**3**). On the other hand, the energetics corresponding to the donation from halogen to B center is significantly high and decreases as 40.7 kcal mol^−1^ for **1**, 35.3 kcal mol^−1^ for **2**, and 30.31 kcal mol^−1^ for **3**. The strength of the donation from the halogen atom is a deciding factor for the P−B and B−X bond lengths. The P−B bond length is shortest for **3** (1.860 Å); this is in agreement with the weaker donation of I lone pairs to the formally empty p‐orbital on B. Note that the strength of the donation from P−C π‐NBO to the B center is also significant viz., 38.4 kcal mol^−1^ for **1**, 49.6 kcal mol^−1^ for **2**, and 56.0 kcal mol^−1^ for **3**. The balance between these second‐order interactions results in the intermediate dihedral angles between the planes defined by terminal B and C atoms with their corresponding substituents (23.5–33.7°).

Based on the geometrical, MO and NBO analyses, the P center in **1**–**3** is monovalent, forming an electron sharing bond with B center while accepting a lone pair of electrons from cAAC. The bonding is schematically represented in Figure 2b. As a result, the P center retains two lone pairs of electrons viz. σ‐ and π‐type. This is a typical description of a carbene stabilized boryl phosphinidene. The singlet boryl phosphinidenes PB(X)(Tipp) (X=Cl, Br, I), with a vacant in‐plane p‐orbital on the P atom, is more stable than the triplet boryl phosphinidenes by 7.94–8.51 kcal mol^−1^ (Figure S22). Hence, the P atom can act as a coordinating center for accepting electrons from cAAC. The calculated dissociation energy of compounds **1**–**3** to respective singlet boryl phosphinidene and cAAC was found to be moderately high, indicative of the strong binding between P−C centers (55.79 kcal mol^−1^ for **1**, 54.60 kcal mol^−1^ for **2**, 53.51 kcal mol^−1^ for **3**). P−C bond dissociation energy values for carbene stabilized phosphinidenes have been calculated to fall in the range 43–75 kcal mol^−1^ at BP86/TZ2P level of theory.^[10]^ From the MO analysis, the π ‐type lone pair on the P center is donated to C←N and B←X π*‐MOs. Accordingly, double Lewis basic behavior can be expected from the P center.

A bent geometry with an active σ‐lone pair and a delocalized π‐MO is the prominent feature of carbones and related species.^[11]^ We computed the proton affinity to assess the basicity of the σ‐lone pair and delocalized π‐MO. The first proton affinity (PA) is quite high (240.7–242.1 kcal mol^−1^) and close to that of NHCs (254.2 kcal mol^−1^) and C(PH_3_)_2_ (255.7 kcal mol^−1^) but less than that in carbodicarbenes, C(NHC^Me^)_2_ (294.3 kcal mol^−1^).^[11a]^ Indeed, the addition of the first proton leads to a tri‐coordinated P center with considerable pyramidalization (34.8°–41.9°; Figure S23), thus indicating that the proton has been added to the delocalized π‐MO in the molecules **1**–**3**. This suggests that the delocalized π‐MO in **1**–**3** is more basic than the σ‐lone pair having a very high s‐character (53.8–54.4 %). Hence, the delocalized π‐MO can be considered as a hidden lone pair. Frenking and co‐workers have used the term hidden lone pair for a delocalized π‐MO in carbodicarbenes and related C(0) compounds, which show very high proton affinity.^[11]^ The PA value is also indicative of its inversely polarized phosphaalkene character.

The first protonation induces an elongation in C_carbene_−P (0.7–1.3 %) and P−B (2.5–2.7 %) bonds and a shortening of the C_carbene_−N bond (2.4–2.6 %; Figure S23). This corroborated well with increased cAAC group charge (0.49–0.52) and decreased negative natural charge on N (−0.35) atom. The MO analysis indicates an active lone pair (35.0–38.2 % s character) with a major coefficient on the P atom (Figure S24). The presence of an active lone pair with relatively low s‐character induces the addition of the second proton at the P center. The calculated second proton affinity is also quite high (137.8–141.9 kcal mol^−1^), indicating that P (I) center in molecules **1**–**3** can act as a double Lewis base.

## Conclusion

In conclusion, we have designed, synthesized, and characterized three cAAC‐stabilized boryl‐phosphinidenes, which also show inversely polarized phosphaalkene characteristics. Quantum mechanical calculations reveal that compounds **1**–**3** feature a P(I) center with two lone pairs, viz. a highly reactive “hidden” π‐lone pair and a σ‐lone pair.

## Experimental Section

**Essential experimental**: Crystal data for **1** at 100(2) K: C_35_H_54_Cl_2_BClNP, *M*_r_=566.02 g/mol, 0.401×0.303×0.139 mm, monoclinic, *P*2_1_/*n*, *a*=9.810(2) Å, *b*=17.822(3) Å, *c*=19.840(3) Å, *β*=92.64(2)°, *V*=3465.0(11) Å^3^, *Z*=4, *μ*(Mo_Kα_)=0.179 mm^−1^, *θ*_max_=25.510°, 78252 reflections measured, 6411 independent (*R*_int_=0.0530), *R*_1_=0.0364 [*I*>2*σ*(*I*)], *wR*_2_=0.0943 (all data), res. density peaks: 0.383 to −0.237 e A^−3^, CCDC: 2058117.

Crystal data for **2** at 100(2) K: C_35_H_54_BBrNP, *M*_**r**_=610.48 g/mol, 0.584×0.317×0.309 mm, triclinic, *P* $\bar{1}$ , *a*=9.805(2) Å, *b*=11.181(2) Å, *c*=16.560(3) Å, *α*=73.82(2)°, *β*=82.35(2)°, *γ*=88.07(3)°, *V*=1728.0(6) Å^3^, *Z*=2, *μ*(Mo_Kα_)=1.258 mm^−1^, *θ*_max_=27.934°, 53463 reflections measured, 8274 independent (*R*_int_=0.0469), *R*_1_=0.0265 [*I*>2*σ*(*I*)], *wR*_2_=0.0674 (all data), res. density peaks: 0.368 to −0.357 e A^−3^.

Crystal data for **3** at 100(2) K: C_42_H_62_BINP, *M*_**r**_=749.60 g/mol, 0.341×0.253×0.235 mm, triclinic, *P* $\bar{1}$ , *a*=10.676(2) Å, *b*=12.167(2) Å, *c*=16.836(3) Å, *α*=107.24(2)°, *β*=94.60(2)°, *γ*=101.62(3)°, *V*=2022.8(7) Å^3^, *Z*=2, *μ*(Mo K_α_)=0.859 mm^−1^, *θ*_max_=27.200°, 82576 reflections measured, 9022 independent (*R*_int_=0.0303), *R*_1_=0.0213 [*I*>2*σ*(*I*)], *wR*_2_=0.0531 (all data), res. density peaks: 0.452 to −0.462 e A^−3^.

All crystals were selected under cooling by using an X‐Temp2 device.^[12]^ The data were integrated with SAINT.^[13]^ A multiscan absorption correction and a 3*λ* correction^[14]^ in **1** was applied using SADABS.^[15]^ The structures were solved by SHELXT^[16]^ and refined on *F* ^2^ using SHELXL^[17]^ in the graphical user interface SHELXLE.^[18]^

Deposition Numbers 2058117 (**1**), 2058118 (**2**), and 2058119 (**3**) contain the supplementary crystallographic data for this paper. These data are provided free of charge by the joint Cambridge Crystallographic Data Centre and Fachinformationszentrum Karlsruhe Access Structures service.

## Conflict of interest

The authors declare no conflict of interest.

1

## Supporting information

As a service to our authors and readers, this journal provides supporting information supplied by the authors. Such materials are peer reviewed and may be re‐organized for online delivery, but are not copy‐edited or typeset. Technical support issues arising from supporting information (other than missing files) should be addressed to the authors.

Supporting Information

## Data Availability

The data that support the findings of this study are available on request from the corresponding author. The data are not publicly available due to privacy or ethical restrictions.
