# Supplementary material for: A Carbene‐Stabilized Boryl‐Phosphinidene
Source: Chemistry. 2022 Apr 13;28(31):e202200913. doi: 10.1002/chem.202200913 (PMC9322276; doi:10.1002/chem.202200913)
Supplement: Supplementary file 1 — Supporting Information [file CHEM-28-0-s001.pdf]

Chemistry–A European Journal

Supporting Information

A Carbene-Stabilized Boryl-Phosphinidene

Samir Kumar Sarkar, Subrata Kundu,* Mohd Nazish, Johannes Kretsch, Regine Herbst-Irmer, Dietmar Stalke,* Parameswaran Parvathy, Pattiyil Parameswaran,* and Herbert W. Roesky*

Table of Contents: **Page Number**

(S1) Materials and Methods (2-3)

(S2) NMR and Mass spectra (4-10)

(S3) X-Ray Crystallographic Analysis (11-20)

(S4) Theoretical calculations (21-30)

(S5) Cartesian coordinates (30-42)

(S6) References (43)

(S1) Materials and Methods

General considerations

All manipulations are carried out using standard Schlenk and glovebox techniques under high purity dinitrogen gas atmosphere. Hexane, toluene and THF are distilled over Na/K alloy (25:75). Deuterated NMR solvent C_6D_6 is dried by stirring for 2 days over Na/K alloy followed by distillation in vacuum and degassed. ^{1}H, ^{13}C, ^{11}B and ^{31}P NMR spectra are recorded on Bruker Avance 300 and 500 MHz NMR spectrometers and referenced to the resonances of the solvent used. Microanalyses are performed by the Analytisches Labor für Anorganische Chemie at the Universität Göttingen. Melting points are determined in sealed glass capillaries under dinitrogen gas atmosphere. LIFDI measurements are performed on a Joel AccuTOF spectrometer under an inert atmosphere. (cAAC)PK was synthesized from the reaction between (cAAC)PH (0.318g, 1.0 mmol) and BzK (0.135g, 1.0 mmol) according to the reported literature procedure.[1] The in-situ prepared (cAAC)PK (0.1 mmol) solution in toluene was used as it is for the further reactions. Trip-BX_2 (X = Cl, Br, I) were synthesized according to the literature procedure,[2] while all other reagents are used as received.

Synthesis of 1 (cAAC=P=B(Cl)Trip) (1):

To a stirring solution of cAACPK (1.0 mmol) at –78 °C in toluene, Trip-BCl_2 (0.285 g; 1.0 mmol) was added. Stirring was continued till the solution came to room temperature and stirring continued for overnight. The solution was then filtered over a G4 frit. The volume of toluene was reduced to 3 mL and the solution was cooled to −35 °C while dark yellow-coloured crystals were formed which were suitable for X-ray diffraction.

Yield: 61 %. Mp: 198 °C. ^{1}H NMR (300 MHz, C_6D_6, 298 K, δ ppm): 7.20-7.00 (m, 5H, Ar-*H*), 3.53 (m, 2H, C*H*Me$_2$), 2.83 (m, 1H, C*H*Me$_2$), 2.71(m, 2H, C*H*Me$_2$), 1.82 (s, 4H, C*Me$_2$*); 1.66 (s, 2H, C*H$_2$*), 1.46 (d, 6H, CH*Me$_2$*, *J* = 6 Hz), 1.39 (d, 6H, CH*Me$_2$*, *J* = 6 Hz), 1.34 (d, 6H, CH*Me$_2$*, *J* = 6 Hz), 1.25 (d, 6H, CH*Me$_2$*, *J* = 6 Hz), 1.16 (d, 6H, CH*Me$_2$*, *J* = 6 Hz), 0.90 (s, 6H, C*Me$_2$*). ^{13}C{^{1}H} NMR (125 MHz, C_6D_6, 298K, ppm): δ = 14.2, 23.8, 24.4, 25.5, 26.8, 28.9, 29.7, 30.4, 31.8, 34.8, 51.2, 53.3, 54.7, 72.0, 120.4, 125.3, 128.3, 129.5, 133.3, 136.7, 146.5, 148.7, 149.0, 220.9 (d, $C_{carbene}$, $J_{c\text{-}p}$ = 91.9 Hz). ^{31}P{^{1}H} NMR (202 MHz, 298 K, C_6D_6) δ +35.5. ^{11}B NMR (160.5 MHz, C_6D_6, 298 K, ppm): δ 72.83. Anal. Calcd (%) for $C_{35}H_{54}BClNP$ (M_w= 566.06): C, 74.27; H, 9.62; N, 2.47. Found: C, 73.03; H, 9.55; N, 2.51.

Synthesis of 2 (cAAC=P=B(Br)Trip) (2):

Compound **2** was synthesized by following the method used for **1**. In this case Trip-BBr_2 (0.375g; 1.0 mmol) was taken instead of Trip-BCl_2.

Yield: 64 %. Mp: 185 °C. ^{1}H NMR (300 MHz, C_6D_6, 298 K, δ ppm): 7.18-7.01 (m, 5H, Ar-*H*), 3.52 (m, 2H, C*H*Me$_2$), 2.83 (m, 1H, C*H*Me$_2$), 2.69 (m, 2H, C*H*Me$_2$), 1.61 (s, 4H, C*Me$_2$*); 1.56 (s, 2H, C*H$_2$*), 1.48 (d, 6H, CH*Me$_2$*, *J* = 6 Hz), 1.45 (d, 6H, CH*Me$_2$*, *J* = 6 Hz), 1.39 (d, 6H, CH*Me$_2$*, *J* = 6 Hz), 1.27 (d, 6H, CH*Me$_2$*, *J* = 9 Hz), 1.15 (d, 6H, CH*Me$_2$*, *J* = 6 Hz), 0.86 (s, 6H, C*Me$_2$*). ^{13}C{^{1}H} NMR (125 MHz, C_6D_6, 298K, ppm): δ = 23.5, 23.8, 24.3, 25.7, 26.7, 28.7, 29.5, 30.9,

34.7, 51.5, 51.6, 54.4, 72.4, 120.6, 125.3, 128.3, 129.6, 133.0, 146.3, 148.4, 149.0, 220.8 (d, $C_{carbene}$, J_{c-p} = 94.5 Hz). $^{31}P\{^1H\}$ NMR (202 MHz, 298 K, C_6D_6) δ +47.1. ^{11}B NMR (160.5 MHz, C_6D_6, 298 K, ppm): δ 71.81. Anal. Calcd (%) for $C_{35}H_{54}BBrNP$ (M_w = 610.51): C, 68.86; H, 8.92; N, 2.29. Found: C, 68.15; H, 8.83; N, 2.35.

Synthesis of 3 (cAAC=P=B(I)Trip) (3):

Compound **3** was synthesized by following the method used for **1.** In this case Trip-BI_2 (0.468g; 1.0 mmol) was taken instead of Trip-BCl_2.

Yield: 58 %. Mp: 178 °C. ^1H NMR (300 MHz, C_6D_6, 298 K, δ ppm): 7.15-6.95 (m, 5H, Ar-*H*), 3.52 (m, 2H, C*H*Me$_2$), 2.84 (m, 1H, C*H*Me$_2$), 2.66 (m, 2H, C*H*Me$_2$), 1.52 (d, 6H, CH*Me$_2$*, *J* = 6 Hz), 1.49 (d, 6H, CH*Me$_2$*, *J* = 6 Hz), 1.46 (s, 2H, C*H$_2$*), 1.41 (d, 6H, CH*Me$_2$*, *J* = 6 Hz), 1.33 (s, 6H, C*Me$_2$*), 1.27 (d, 6H, CH*Me$_2$*, *J* = 6 Hz), 1.15 (d, 6H, CH*Me$_2$*, *J* = 6 Hz), 0.82 (s, 6H, C*Me$_2$*). $^{13}C\{^1H\}$ NMR (C_6D_6, 298K, ppm): δ = 23.0, 23.8, 24.3, 25.4, 26.7, 26.8, 28.5, 29.6, 31.1, 34.6, 34.7, 51.6, 51.7, 53.8, 72.8, 121.1, 125.3, 129.8, 132.6, 146.0, 147.5, 149.3, 220.7 (d, $C_{carbene}$, J_{c-p} = 104.6 Hz). $^{31}P\{^1H\}$ NMR (202 MHz, 298 K, C_6D_6) δ +66.9. ^{11}B NMR (160.5 MHz, C_6D_6, 298 K, ppm): δ 69.80. Anal. Calcd (%) for $C_{35}H_{54}BINP \cdot C_7H_8$ (M_w = 750.66): C, 67.20; H, 8.46; N, 1.87. Found: C, 66.77; H, 8.31; N, 1.92.

(S2) NMR and Mass spectra:

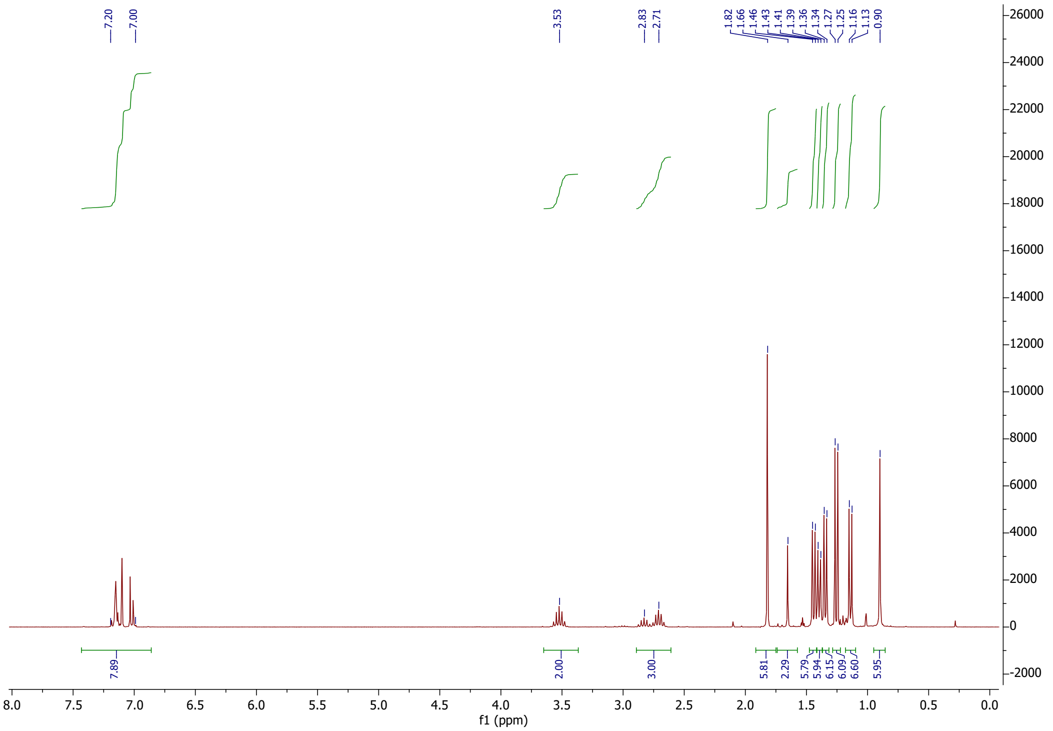

Figure S1. ^{1}H-NMR spectrum of **1**.

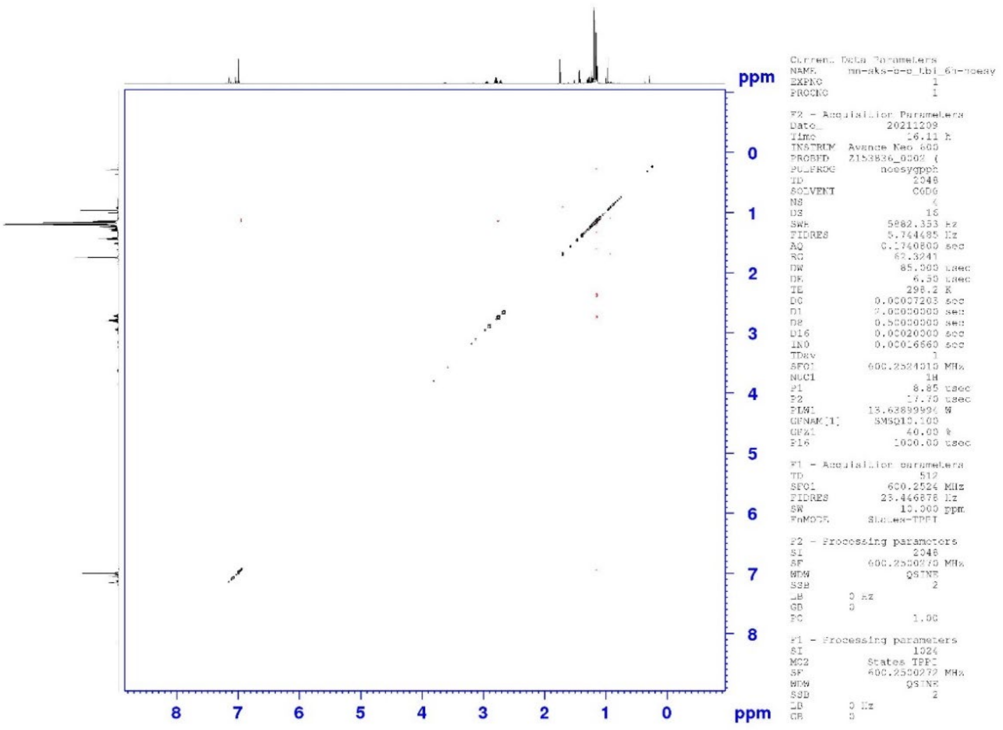

Figure S2. ^{1}H-NOESY NMR spectrum of **1**.

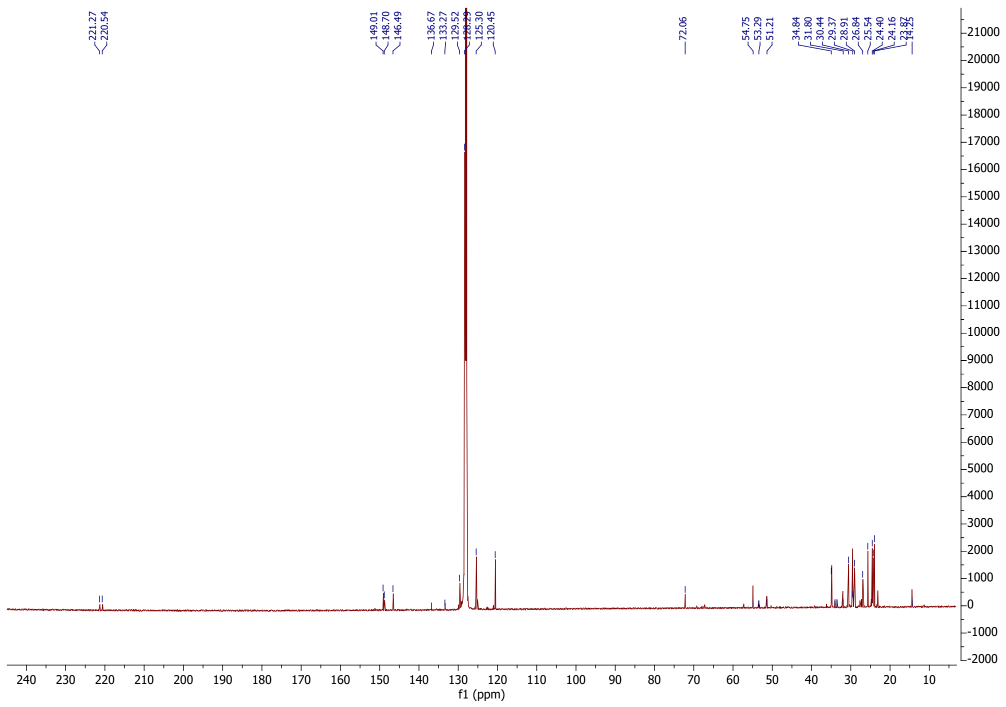

Figure S3. ^{13}C-NMR spectrum of **1**.

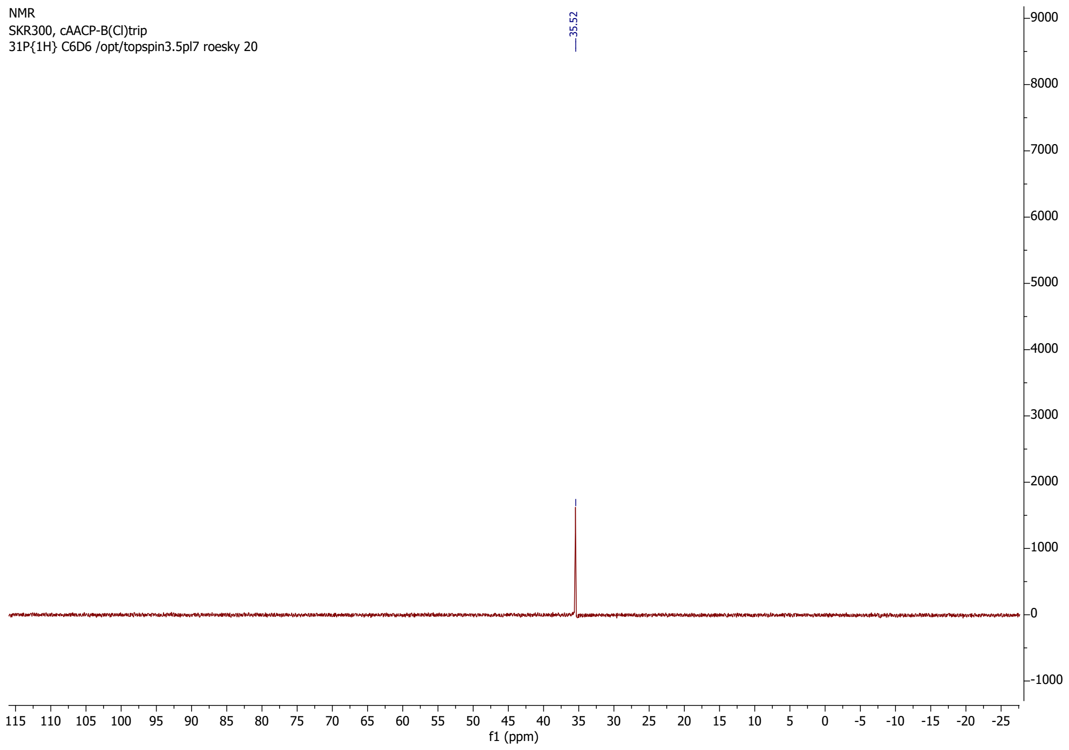

Figure S4. ^{31}P-NMR spectrum of **1**.

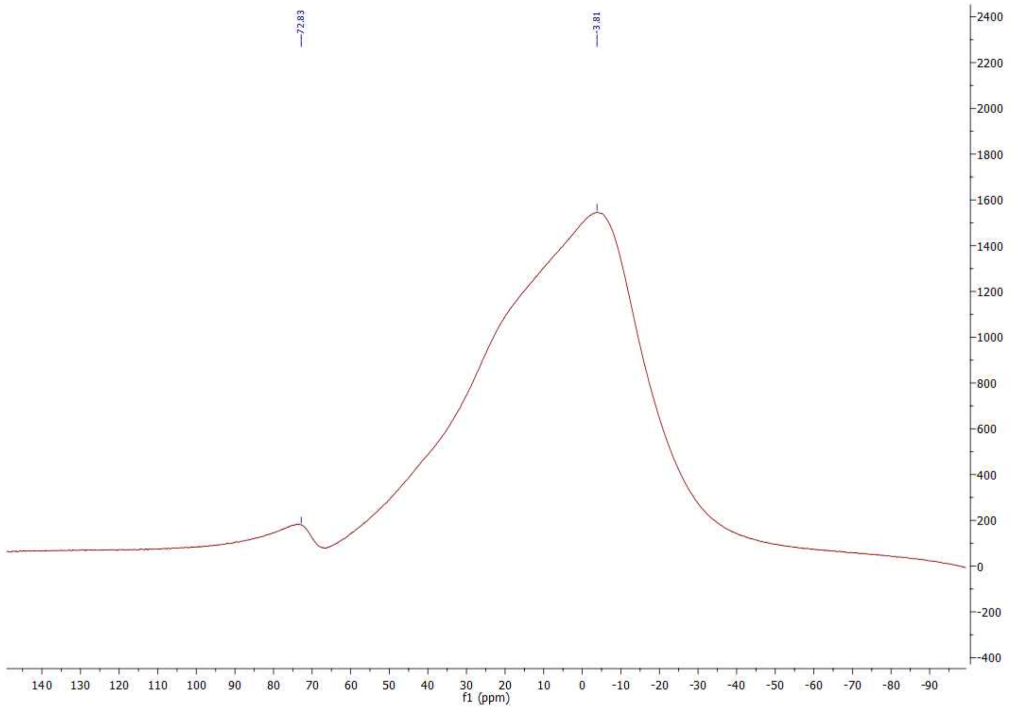

Figure S5. ^{11}B-NMR spectrum of **1**.

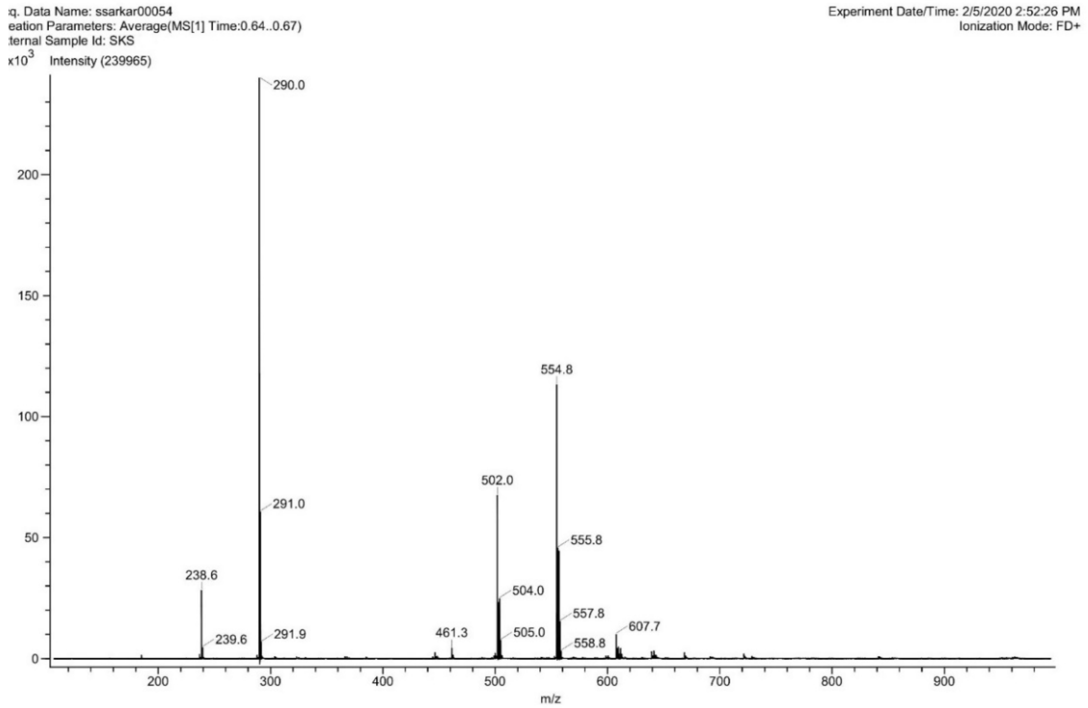

Figure S6. Mass spectrum of **1 [(1-Cl) +Na]**.

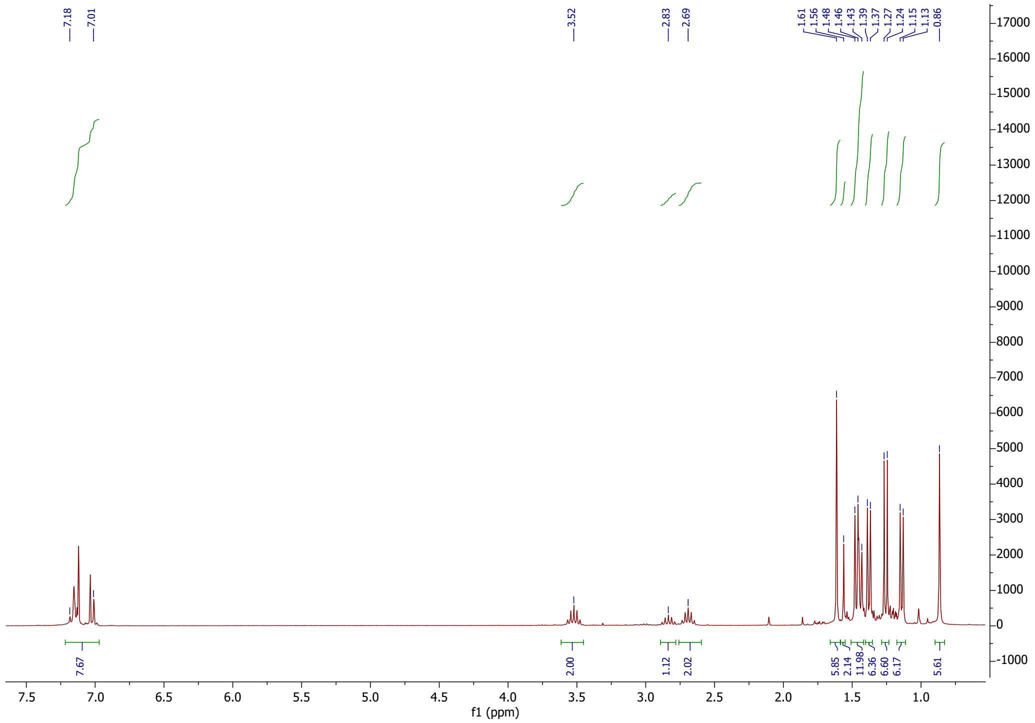

Figure S7. ^{1}H-NMR spectrum of **2**.

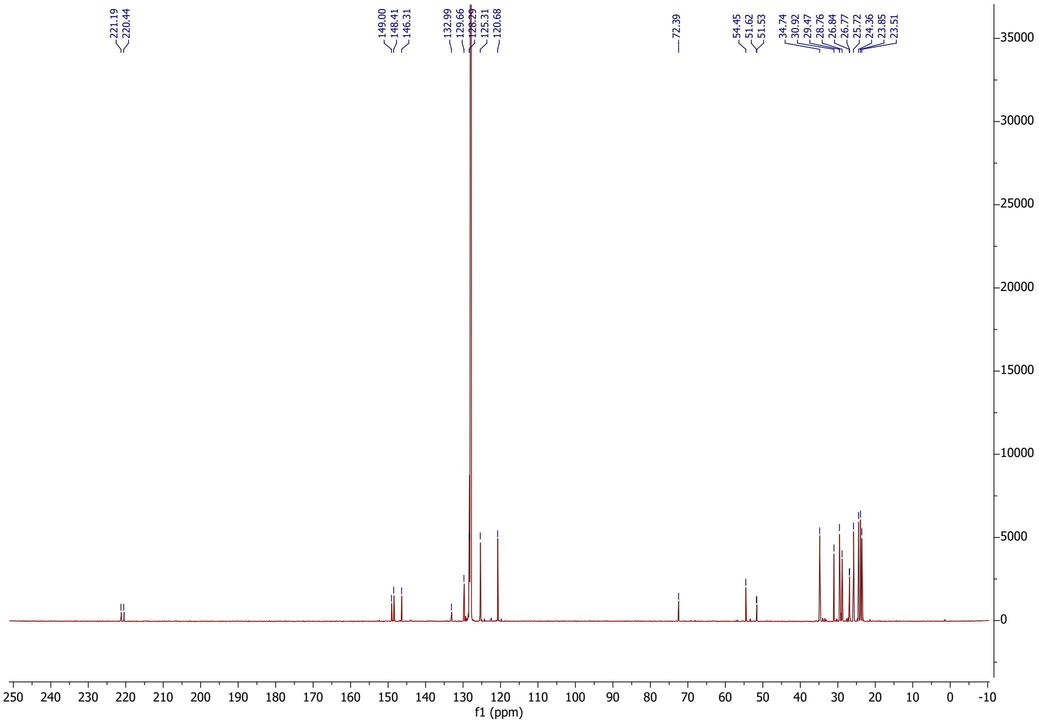

Figure S8. ^{13}C-NMR spectrum of **2**.

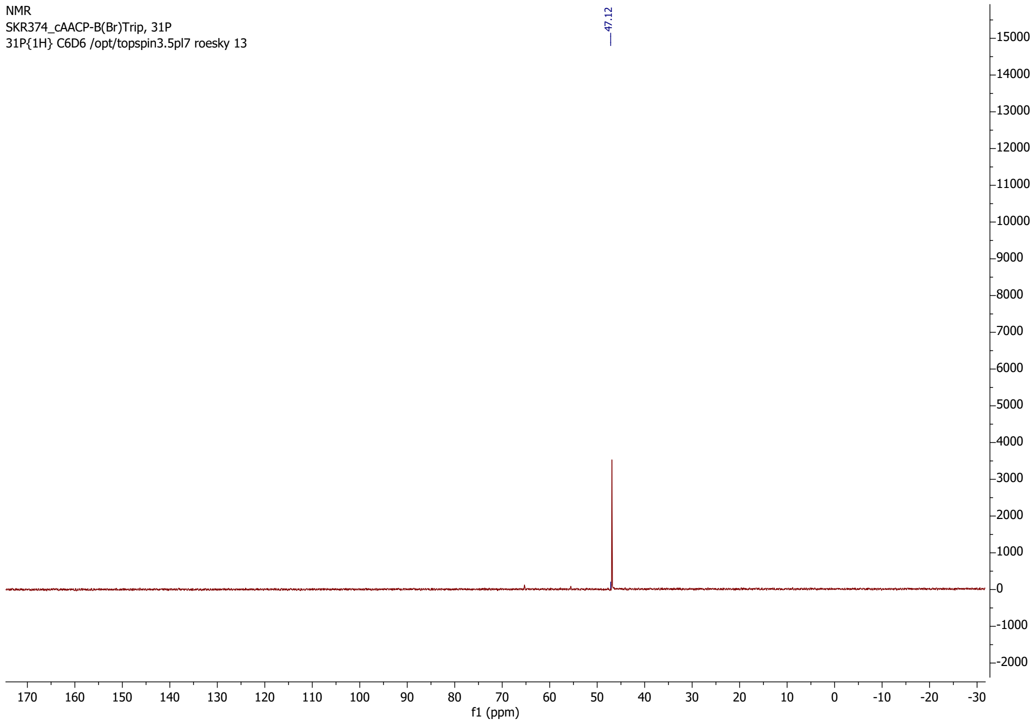

Figure S9. ^{31}P-NMR spectrum of **2**.

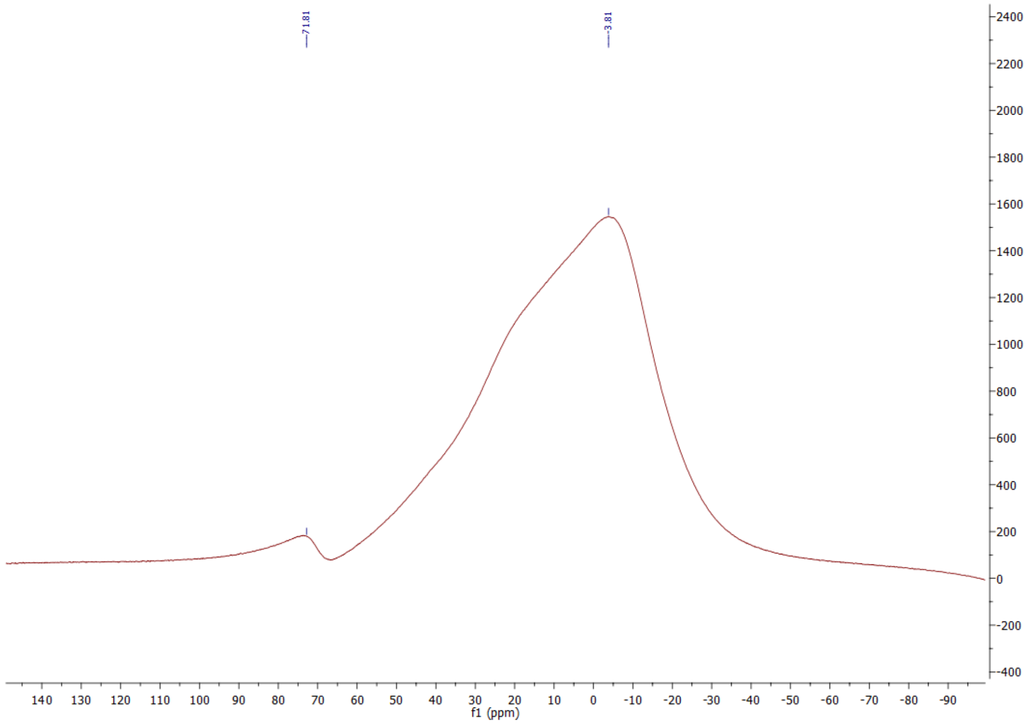

Figure S10. ^{11}B-NMR spectrum of **2**.

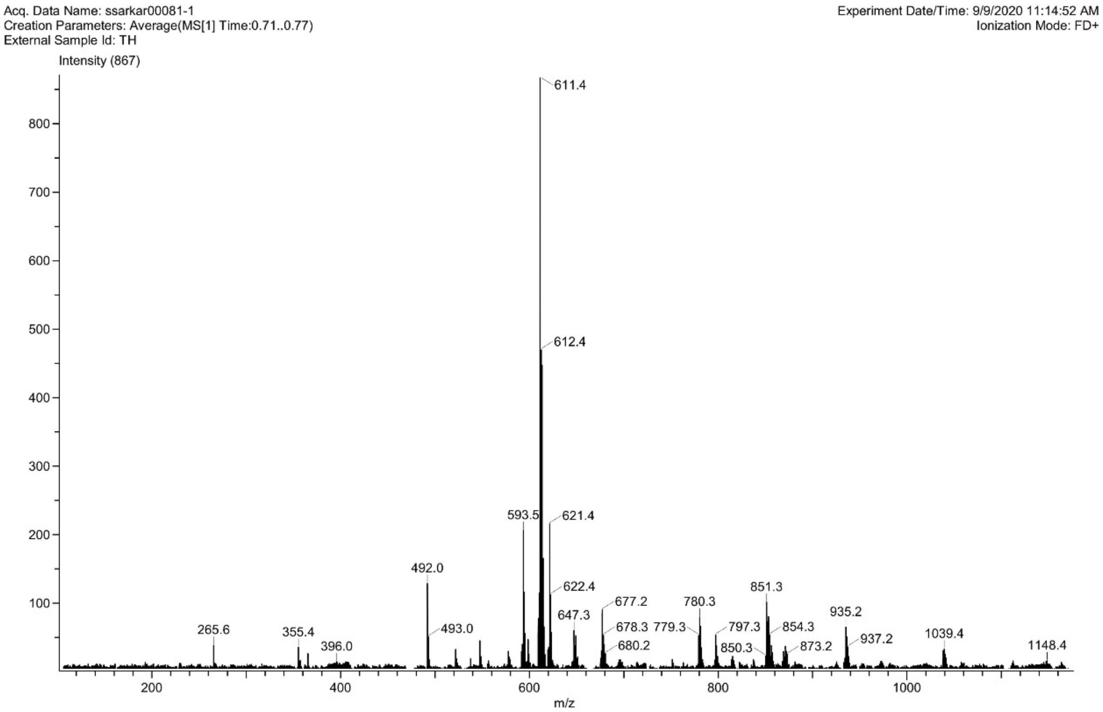

Figure S11. LIFDI-Mass spectrum of **2**.

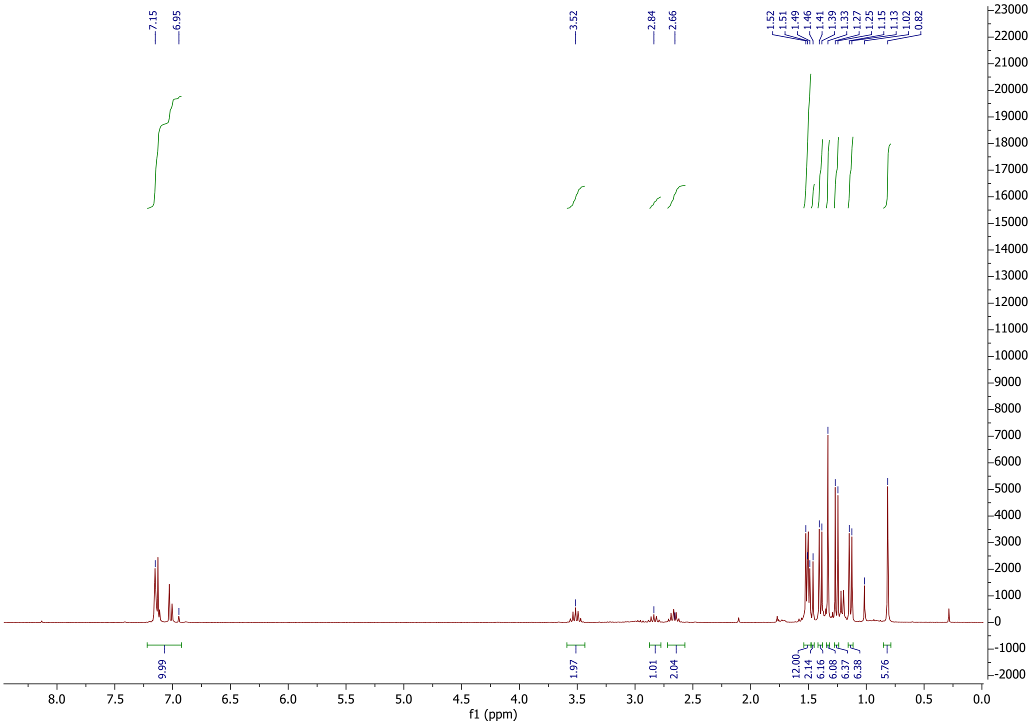

Figure S12. ^{1}H spectra of **3**.

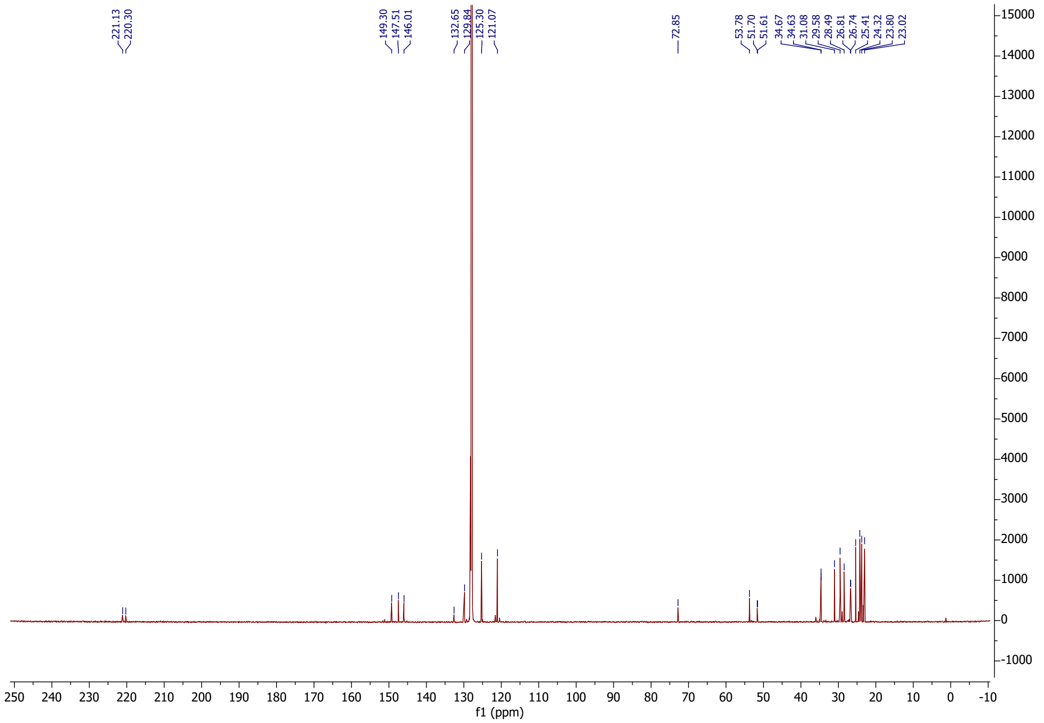

Figure S13. ${}^{13}C\{{}^{1}H\}$ spectra of **3**.

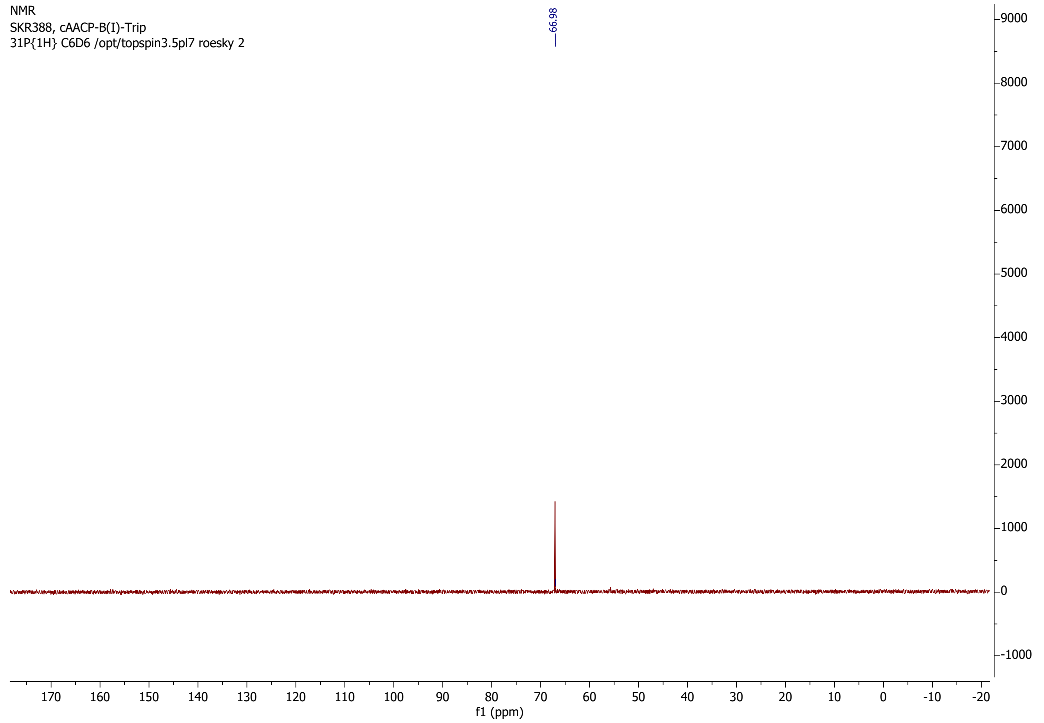

Figure S14. ${}^{31}P\{1H\}$ spectra of **3**.

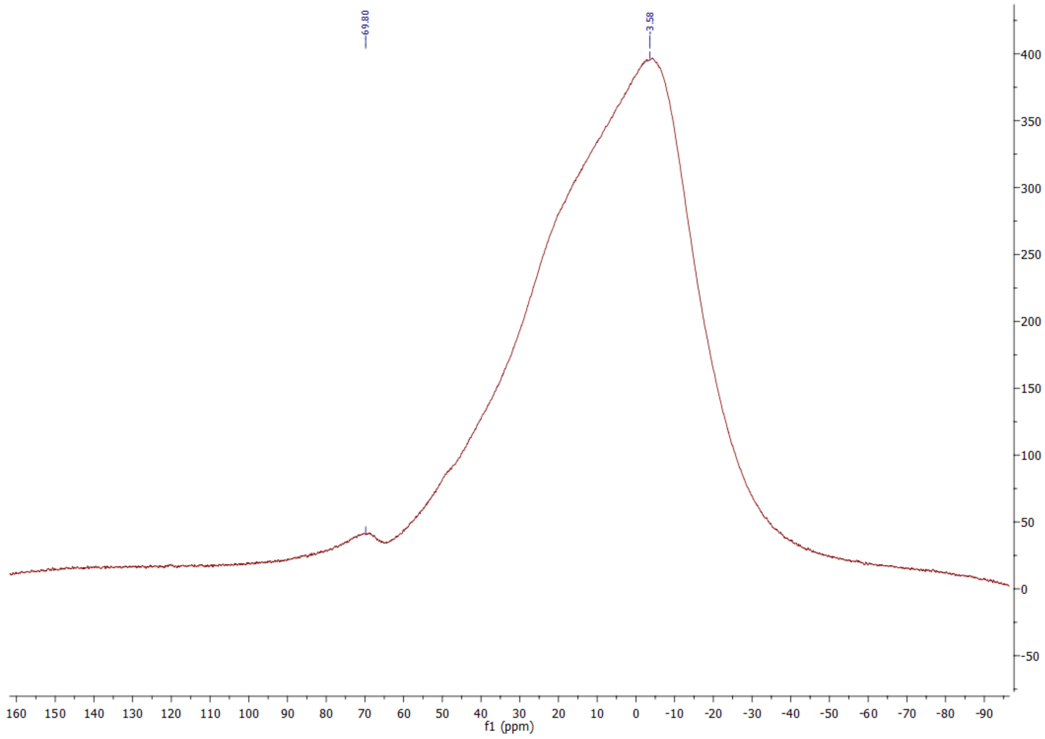

Figure S15. ^{11}B-NMR spectrum of **3**.

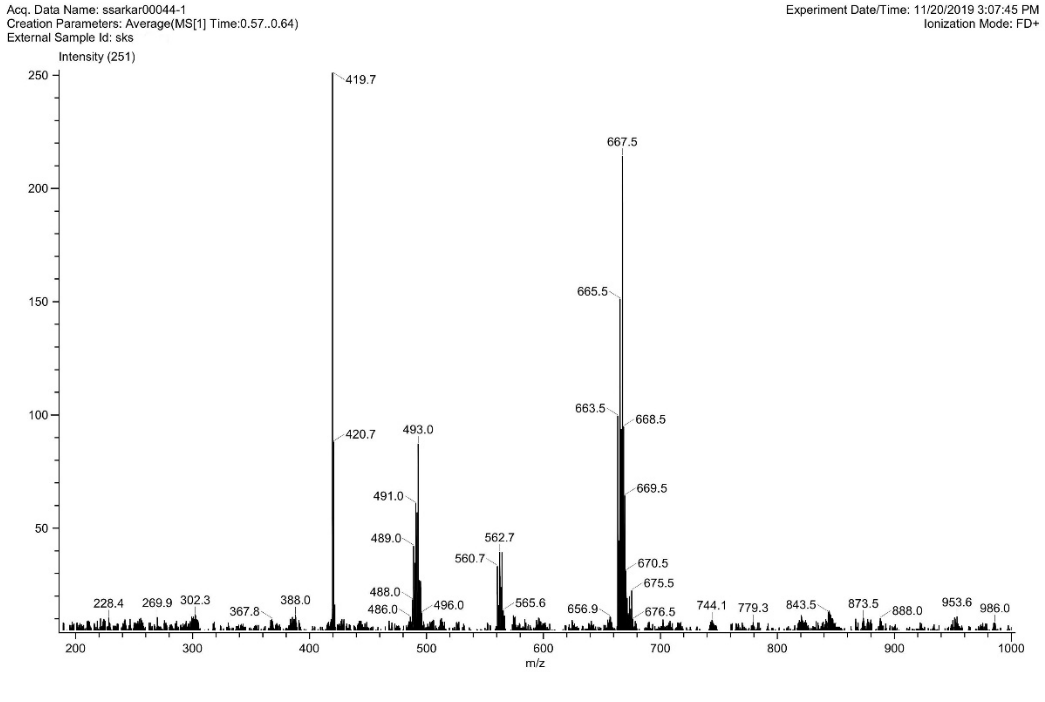

Figure S16. Mass spectrum of **3**.

(S3) Crystallographic Details: Experimental Section:

Crystal data for **1** at 100(2) K: $C_{35}H_{54}Cl_2BClNP$, M_r = 566.02 g/mol, 0.401 × 0.303 × 0.139 mm, monoclinic, $P2_1/n$, a = 9.810(2) Å, b = 17.822(3) Å, c = 19.840(3) Å, β = 92.64(2)°, V = 3465.0(11) Å^3, Z = 4, μ(Mo K_α) = 0.179 mm^{-1}, θ_{max} = 25.510°, 78252 reflections measured, 6411 independent (R_{int} = 0.0530), R_1 = 0.0364 [$I > 2\sigma(I)$], wR_2 = 0.0943 (all data), res. density peaks: 0.383 to −0.237 eA^{-3}, CCDC: 2058117.

Crystal data for **2** at 100(2) K: $C_{35}H_{54}BBrNP$, M_r = 610.48 g/mol, 0.584 × 0.317 × 0.309 mm, triclinic, $P\bar{1}$, a = 9.805(2) Å, b = 11.181(2) Å, c = 16.560(3) Å, α = 73.82(2)°, β = 82.35(2)°, γ = 88.07(3)°, V = 1728.0(6) Å^3, Z = 2, μ(Mo K_α) = 1.258 mm^{-1}, θ_{max} = 27.934°, 53463 reflections measured, 8274 independent (R_{int} = 0.0469), R_1 = 0.0265 [$I > 2\sigma(I)$], wR_2 = 0.0674 (all data), res. density peaks: 0.368 to −0.357 eA^{-3}, CCDC: 2058118.

Crystal data for **3** at 100(2) K: $C_{42}H_{62}BINP$, M_r = 749.60 g/mol, 0.341 × 0.253 × 0.235 mm, triclinic, $P\bar{1}$, a = 10.676(2) Å, b = 12.167(2) Å, c = 16.836(3) Å, α = 107.24(2)°, β = 94.60(2)°, γ = 101.62(3)°, V = 2022.8(7) Å^3, Z = 2, μ(Mo K_α) = 0.859 mm^{-1}, θ_{max} = 27.200°, 82576 reflections measured, 9022 independent (R_{int} = 0.0303), R_1 = 0.0213 [$I > 2\sigma(I)$], wR_2 = 0.0531 (all data), res. density peaks: 0.452 to −0.462 eA^{-3}, CCDC: 2058119.

All crystals were selected under cooling, using a X-Temp2 device[3] The data were integrated with SAINT.[4] A multi-scan absorption correction and a 3λ correction[5] in 1 was applied using SADABS.[6] The structures were solved by SHELXT[7] and refined on F^2 using SHELXL[8] in the graphical user interface SHELXLE.[9]

	**1**	**2**	**3**
Empirical formula	$C_{35}H_{54}$ B Cl N P	$C_{35}H_{54}$ B Br N P	$C_{42}H_{62}$ B I N P
CCDC number	2058117	2058118	2058119
Formula weight	566.02	610.48	749.60
Temperature [K]	100(2)	100(2)	100(2)
Wavelength [Å]	0.71073	0.71073	0.71073
Crystal system	Monoclinic	Triclinic	Triclinic
Space group	$P2_1/n$	$P\bar{1}$	$P\bar{1}$
a [Å]	9.810(2)	9.805(2)	10.676(2)
b [Å]	17.822(3)	11.181(2)	12.167(2)
c [Å]	19.840(3)	16.560(3)	16.836(3)
α [°]	90	73.82(2)	107.24(2)
β [°]	92.64(2)	82.35(2)	94.60(2)
γ [°]	90	88.07(3)	101.62(3)
V [Å^3]	3465.0(11)	1728.0(6)	2022.8(7)
Z	4	2	2
ρ [Mgm^{-3}]	1.085	1.173	1.231
μ [mm^{-1}]	0.179	1.258	0.859
F (000)	1232	652	788

Crystal size [mm]	0.401 × 0.303 × 0.139	0.548 × 0.317 × 0.309	0.341 × 0.253 × 0.235
θ-area [°]	2.055 to 25.510	1.896 to 27.934	1.281 to 27.200
Index ranges	$-11 \le h \le 11$,<br>$-21 \le k \le 21$,<br>$-24 \le l \le 23$	$-12 \le h \le 12$,<br>$-14 \le k \le 14$,<br>$-21 \le l \le 21$	$-13 \le h \le 13$,<br>$-15 \le k \le 15$,<br>$-21 \le l \le 21$
Total number reflect.	78252	53463	82576
Unique reflections	6411	8274	9022
R_{int}	0.0530	0.0469	0.0303
Max. and min. transmission	0.7452 and 0.6767	0.4308 and 0.3701	0.7455 and 0.6758
Data / restraints / parameters	6411 / 73 / 385	8274 / 0 / 366	9022 / 213 / 494
Goodness-of-fit on F^2	1.026	1.035	1.035
$R1$ [$I>2\sigma(I)$]	0.0364	0.0265	0.0213
$wR2$ [$I>2\sigma(I)$]	0.0859	0.0650	0.0518
$R1$ [all data]	0.0505	0.0317	0.0235
$wR2$ [all data]	0.0943	0.0673	0.0531
Largest diff. peak and hole max. / min. [e·Å $^{-3}$]	0.383 and -0.237	0.368 and -0.357	0.452 and -0.462

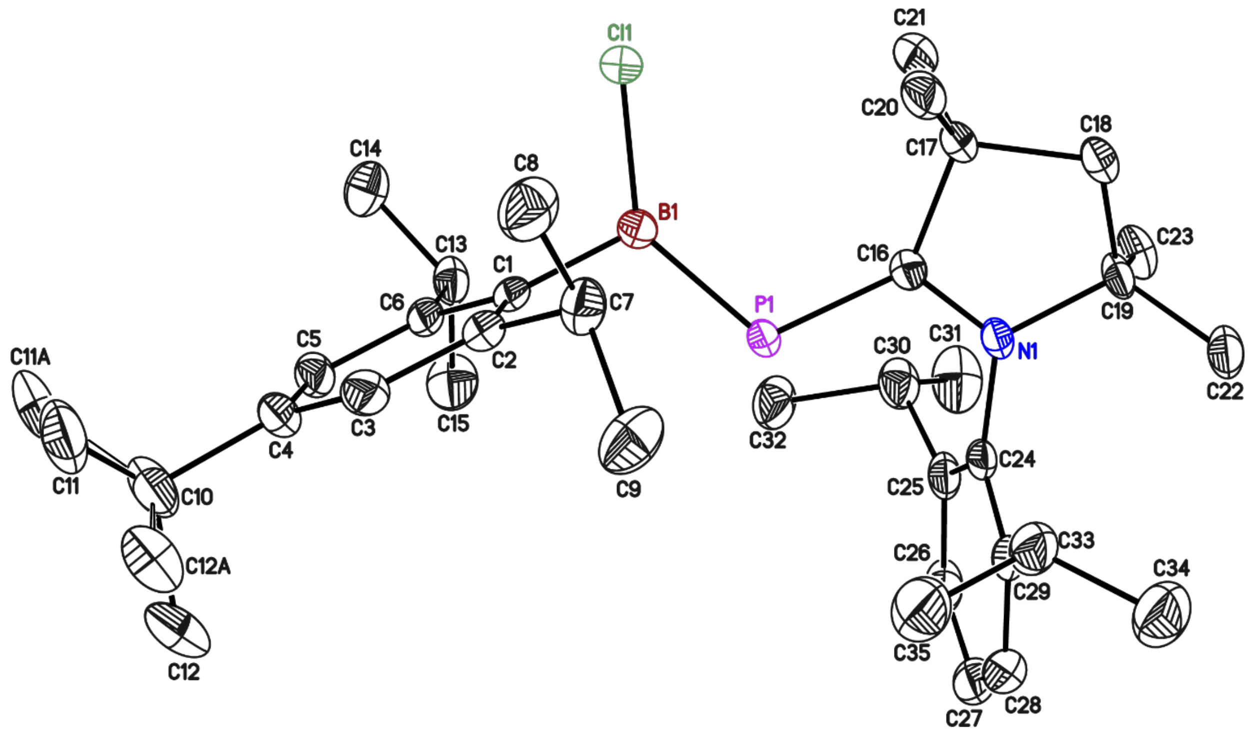

Figure S17. Asymmetric unit of **1**. Displacement parameters are depicted at 50% probability. Hydrogen atoms are omitted for clarity. One of the *iso*-propyl groups is disordered about two positions. It is refined with distance restraints and some of the anisotropic displacement parameters are constrained to be the same. The occupancy of the minor position refined to 0.034(3).

Table S1. Bond lengths [Å] and angles [°] of **1**.

P(1)-C(16)	1.7709(15)	C(5)-C(6)	1.394(2)
P(1)-B(1)	1.8607(18)	C(6)-C(13)	1.517(2)
B(1)-C(1)	1.578(2)	C(7)-C(9)	1.519(3)
B(1)-Cl(1)	1.8046(18)	C(7)-C(8)	1.534(2)
N(1)-C(16)	1.3352(19)	C(4)-C(10)	1.521(2)
N(1)-C(24)	1.4503(19)	C(10)-C(11A)	1.483(16)
N(1)-C(19)	1.5158(18)	C(10)-C(12)	1.505(3)
C(1)-C(2)	1.406(2)	C(10)-C(12A)	1.516(16)
C(1)-C(6)	1.407(2)	C(10)-C(11)	1.516(3)
C(2)-C(3)	1.396(2)	C(13)-C(14)	1.525(2)
C(2)-C(7)	1.522(2)	C(13)-C(15)	1.526(2)
C(3)-C(4)	1.393(2)	C(16)-C(17)	1.526(2)
C(5)-C(4)	1.384(2)	C(17)-C(20)	1.530(2)

C(17)-C(21)	1.532(2)	C(1)-C(6)-C(13)	120.86(13)
C(17)-C(18)	1.545(2)	C(9)-C(7)-C(2)	110.60(14)
C(18)-C(19)	1.528(2)	C(9)-C(7)-C(8)	110.43(15)
C(19)-C(23)	1.521(2)	C(2)-C(7)-C(8)	112.52(13)
C(19)-C(22)	1.521(2)	C(5)-C(4)-C(3)	117.86(14)
C(24)-C(25)	1.405(2)	C(5)-C(4)-C(10)	120.29(16)
C(24)-C(29)	1.408(2)	C(3)-C(4)-C(10)	121.80(16)
C(25)-C(26)	1.398(2)	C(11A)-C(10)-C(12A)	109(2)
C(25)-C(30)	1.517(3)	C(12)-C(10)-C(11)	112.61(19)
C(26)-C(27)	1.372(3)	C(11A)-C(10)-C(4)	114.6(16)
C(27)-C(28)	1.371(3)	C(12)-C(10)-C(4)	110.15(16)
C(28)-C(29)	1.395(2)	C(12A)-C(10)-C(4)	107.7(16)
C(29)-C(33)	1.513(3)	C(11)-C(10)-C(4)	113.44(16)
C(30)-C(31)	1.532(3)	C(6)-C(13)-C(14)	111.29(13)
C(30)-C(32)	1.543(2)	C(6)-C(13)-C(15)	112.51(14)
C(33)-C(35)	1.535(2)	C(14)-C(13)-C(15)	110.69(14)
C(33)-C(34)	1.536(3)	N(1)-C(16)-C(17)	109.16(12)
		N(1)-C(16)-P(1)	118.45(11)
C(16)-P(1)-B(1)	114.68(7)	C(17)-C(16)-P(1)	132.11(11)
C(1)-B(1)-Cl(1)	112.64(11)	C(16)-C(17)-C(20)	111.18(13)
C(1)-B(1)-P(1)	113.58(11)	C(16)-C(17)-C(21)	110.27(13)
Cl(1)-B(1)-P(1)	133.49(10)	C(20)-C(17)-C(21)	111.70(14)
C(16)-N(1)-C(24)	124.26(12)	C(16)-C(17)-C(18)	102.93(12)
C(16)-N(1)-C(19)	115.22(12)	C(20)-C(17)-C(18)	110.12(13)
C(24)-N(1)-C(19)	120.45(12)	C(21)-C(17)-C(18)	110.32(13)
C(2)-C(1)-C(6)	118.99(14)	C(19)-C(18)-C(17)	107.78(12)
C(2)-C(1)-B(1)	120.31(14)	N(1)-C(19)-C(23)	110.13(12)
C(6)-C(1)-B(1)	120.70(14)	N(1)-C(19)-C(22)	112.11(13)
C(3)-C(2)-C(1)	119.51(15)	C(23)-C(19)-C(22)	108.38(13)
C(3)-C(2)-C(7)	119.80(15)	N(1)-C(19)-C(18)	100.40(11)
C(1)-C(2)-C(7)	120.69(14)	C(23)-C(19)-C(18)	114.19(14)
C(4)-C(3)-C(2)	121.91(16)	C(22)-C(19)-C(18)	111.54(13)
C(4)-C(5)-C(6)	122.09(15)	C(25)-C(24)-C(29)	122.07(15)
C(5)-C(6)-C(1)	119.63(15)	C(25)-C(24)-N(1)	119.49(15)
C(5)-C(6)-C(13)	119.51(14)	C(29)-C(24)-N(1)	118.43(14)

C(26)-C(25)-C(24)	117.15(17)	C(24)-C(29)-C(33)	123.84(15)
C(26)-C(25)-C(30)	118.45(16)	C(25)-C(30)-C(31)	113.28(15)
C(24)-C(25)-C(30)	124.21(14)	C(25)-C(30)-C(32)	108.84(15)
C(27)-C(26)-C(25)	121.57(19)	C(31)-C(30)-C(32)	109.62(15)
C(28)-C(27)-C(26)	120.15(17)	C(29)-C(33)-C(35)	110.03(16)
C(27)-C(28)-C(29)	121.64(19)	C(29)-C(33)-C(34)	113.11(16)
C(28)-C(29)-C(24)	117.15(17)	C(35)-C(33)-C(34)	108.97(16)
C(28)-C(29)-C(33)	118.83(17)		

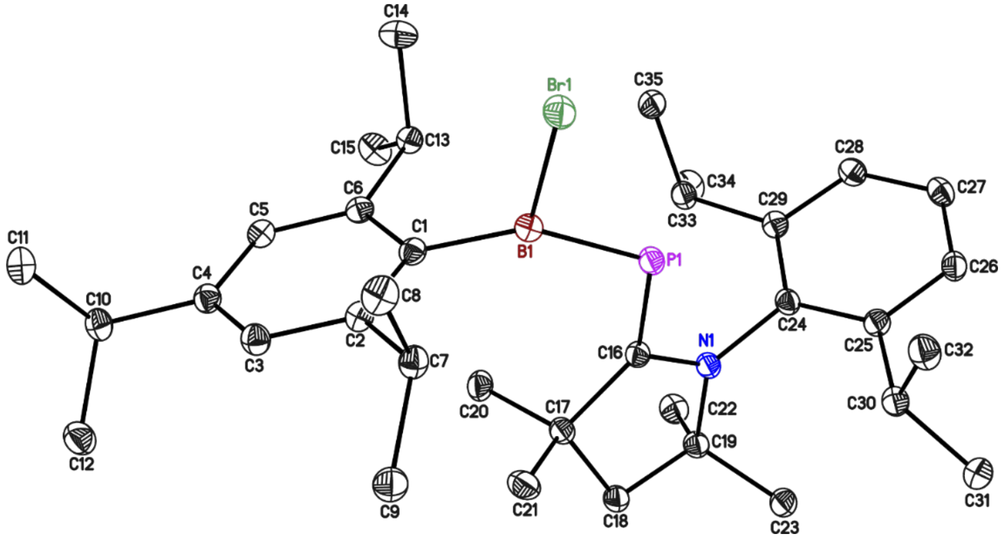

Figure S18. Asymmetric unit of **2**. Displacement parameters are depicted at 50% probability. Hydrogen atoms are omitted for clarity.

Table S2. Bond lengths [Å] and angles [°] of **2**.

Br(1)-B(1)	2.0085(15)	C(10)-C(11)	1.535(2)
C(1)-C(2)	1.4144(17)	C(10)-C(12)	1.536(2)
C(1)-C(6)	1.4164(18)	C(13)-C(14)	1.5296(19)
C(1)-B(1)	1.5744(18)	C(13)-C(15)	1.5314(19)
N(1)-C(16)	1.3379(16)	C(16)-C(17)	1.5338(17)
N(1)-C(24)	1.4559(15)	C(17)-C(21)	1.5325(19)
N(1)-C(19)	1.5144(16)	C(17)-C(20)	1.5393(19)
B(1)-P(1)	1.8607(15)	C(17)-C(18)	1.5422(18)
P(1)-C(16)	1.7850(14)	C(18)-C(19)	1.5319(18)
C(4)-C(3)	1.3919(19)	C(19)-C(23)	1.5249(19)
C(4)-C(5)	1.3952(18)	C(19)-C(22)	1.530(2)
C(4)-C(10)	1.5222(17)	C(24)-C(25)	1.4105(18)
C(3)-C(2)	1.3945(18)	C(24)-C(29)	1.4136(18)
C(2)-C(7)	1.5257(18)	C(25)-C(26)	1.3976(18)
C(6)-C(5)	1.3974(18)	C(25)-C(30)	1.5277(19)
C(6)-C(13)	1.5281(17)	C(26)-C(27)	1.3873(19)
C(9)-C(7)	1.5391(19)	C(27)-C(28)	1.3824(19)
C(8)-C(7)	1.539(2)	C(28)-C(29)	1.3977(18)

C(29)-C(33)	1.5233(19)
C(30)-C(32)	1.539(2)
C(30)-C(31)	1.541(2)
C(33)-C(35)	1.533(2)
C(33)-C(34)	1.537(2)
C(2)-C(1)-C(6)	118.89(11)
C(2)-C(1)-B(1)	119.83(11)
C(6)-C(1)-B(1)	121.27(11)
C(16)-N(1)-C(24)	124.28(11)
C(16)-N(1)-C(19)	115.26(10)
C(24)-N(1)-C(19)	120.31(10)
C(1)-B(1)-P(1)	141.65(10)
C(1)-B(1)-Br(1)	112.98(9)
P(1)-B(1)-Br(1)	105.20(7)
C(16)-P(1)-B(1)	111.93(6)
C(3)-C(4)-C(5)	117.96(12)
C(3)-C(4)-C(10)	120.95(11)
C(5)-C(4)-C(10)	121.07(12)
C(4)-C(3)-C(2)	122.02(12)
C(3)-C(2)-C(1)	119.63(12)
C(3)-C(2)-C(7)	119.26(11)
C(1)-C(2)-C(7)	121.07(11)
C(5)-C(6)-C(1)	119.39(11)
C(5)-C(6)-C(13)	120.30(11)
C(1)-C(6)-C(13)	120.28(11)
C(4)-C(5)-C(6)	121.99(12)
C(2)-C(7)-C(8)	109.94(11)
C(2)-C(7)-C(9)	113.76(11)
C(8)-C(7)-C(9)	109.68(11)
C(4)-C(10)-C(11)	110.38(11)
C(4)-C(10)-C(12)	111.57(11)
C(11)-C(10)-C(12)	111.40(12)
C(6)-C(13)-C(14)	110.43(11)
C(6)-C(13)-C(15)	113.91(11)
C(14)-C(13)-C(15)	109.56(11)
N(1)-C(16)-C(17)	108.72(11)
N(1)-C(16)-P(1)	117.41(9)
C(17)-C(16)-P(1)	133.70(9)
C(21)-C(17)-C(16)	112.47(11)
C(21)-C(17)-C(20)	109.86(11)
C(16)-C(17)-C(20)	110.27(11)
C(21)-C(17)-C(18)	110.21(11)
C(16)-C(17)-C(18)	102.74(10)
C(20)-C(17)-C(18)	111.13(11)
C(19)-C(18)-C(17)	107.43(10)
N(1)-C(19)-C(23)	110.89(11)
N(1)-C(19)-C(22)	111.43(11)
C(23)-C(19)-C(22)	108.29(11)
N(1)-C(19)-C(18)	100.54(10)
C(23)-C(19)-C(18)	111.85(11)
C(22)-C(19)-C(18)	113.73(11)
C(25)-C(24)-C(29)	121.64(11)
C(25)-C(24)-N(1)	119.65(11)
C(29)-C(24)-N(1)	118.67(11)
C(26)-C(25)-C(24)	117.47(12)
C(26)-C(25)-C(30)	118.12(12)
C(24)-C(25)-C(30)	124.23(11)
C(27)-C(26)-C(25)	121.93(12)
C(28)-C(27)-C(26)	119.21(12)
C(27)-C(28)-C(29)	121.95(12)
C(28)-C(29)-C(24)	117.53(12)
C(28)-C(29)-C(33)	118.74(12)
C(24)-C(29)-C(33)	123.64(11)
C(25)-C(30)-C(32)	109.76(11)
C(25)-C(30)-C(31)	112.72(12)
C(32)-C(30)-C(31)	109.05(12)
C(29)-C(33)-C(35)	110.86(11)
C(29)-C(33)-C(34)	111.89(12)
C(35)-C(33)-C(34)	109.30(12)

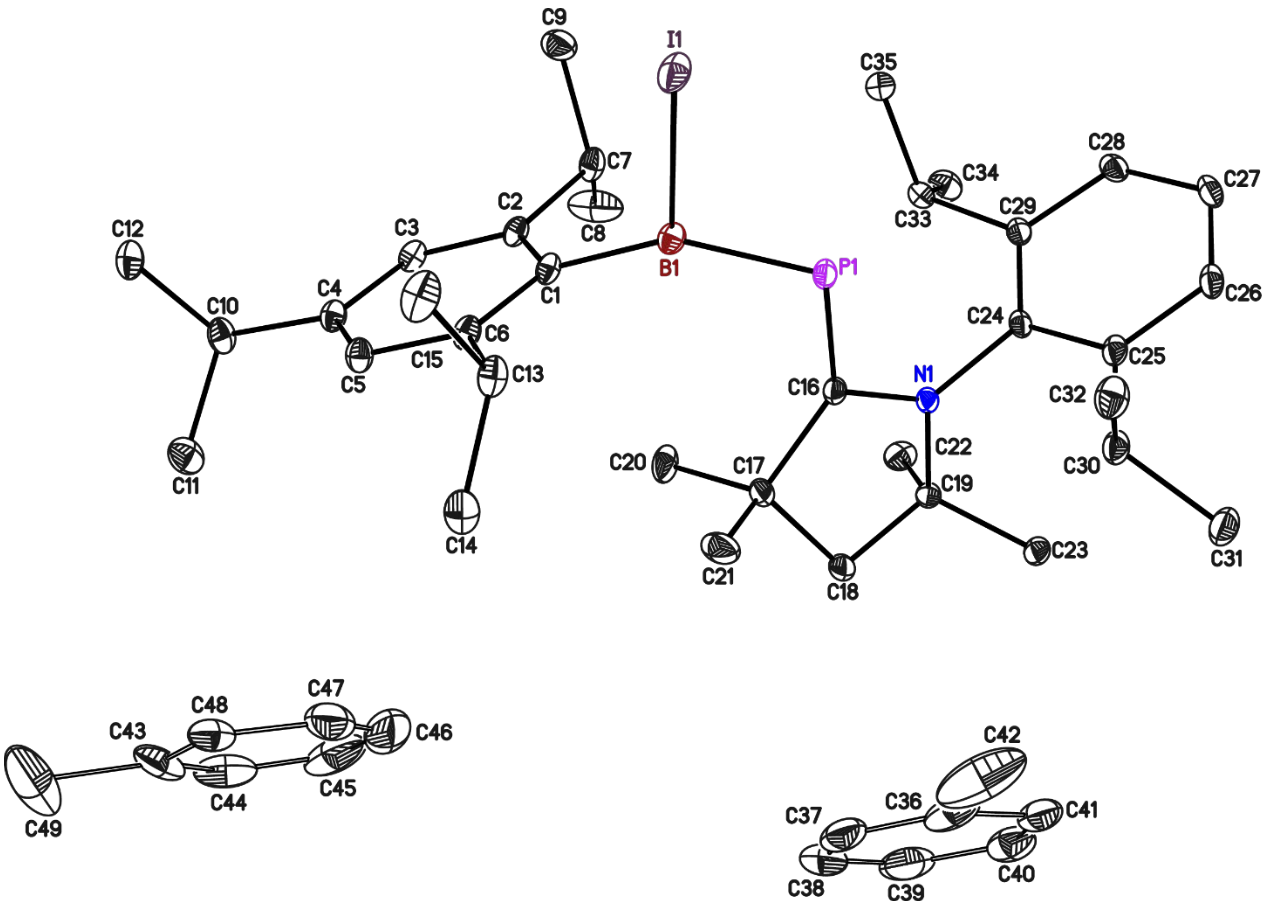

Figure S19. Asymmetric unit of **3**. Displacement parameters are depicted at 50% probability. Hydrogen atoms are omitted for clarity. The toluene molecules C36 to C42 and C43 to C49 are disordered about an inversion center.

Table S3. Bond lengths [Å] and angles [°] for **3**.

I(1)-B(1)	2.2504(15)	C(4)-C(5)	1.3969(19)
C(1)-C(6)	1.4145(18)	C(4)-C(10)	1.5170(18)
C(1)-C(2)	1.4160(18)	C(3)-C(2)	1.3922(18)
C(1)-B(1)	1.5706(19)	C(2)-C(7)	1.5238(18)
N(1)-C(16)	1.3306(16)	C(5)-C(6)	1.3932(18)
N(1)-C(24)	1.4562(15)	C(6)-C(13)	1.5277(19)
N(1)-C(19)	1.5204(16)	C(7)-C(9)	1.520(2)
P(1)-C(16)	1.7944(14)	C(7)-C(8)	1.526(2)
P(1)-B(1)	1.8456(15)	C(10)-C(11)	1.527(2)
C(4)-C(3)	1.3885(19)	C(10)-C(12)	1.528(2)

C(14)-C(13)	1.534(2)		
C(13)-C(15)	1.535(2)	C(6)-C(1)-C(2)	118.46(11)
C(16)-C(17)	1.5376(18)	C(6)-C(1)-B(1)	121.17(12)
C(17)-C(21)	1.530(2)	C(2)-C(1)-B(1)	120.35(12)
C(17)-C(20)	1.537(2)	C(16)-N(1)-C(24)	124.53(10)
C(17)-C(18)	1.5433(19)	C(16)-N(1)-C(19)	114.62(10)
C(18)-C(19)	1.5271(18)	C(24)-N(1)-C(19)	120.84(10)
C(19)-C(23)	1.5225(18)	C(16)-P(1)-B(1)	110.77(6)
C(19)-C(22)	1.527(2)	C(1)-B(1)-P(1)	142.65(10)
C(24)-C(29)	1.4034(19)	C(1)-B(1)-I(1)	112.49(9)
C(24)-C(25)	1.4131(19)	P(1)-B(1)-I(1)	104.32(7)
C(25)-C(26)	1.3970(18)	C(3)-C(4)-C(5)	117.85(12)
C(25)-C(30)	1.524(2)	C(3)-C(4)-C(10)	120.57(12)
C(26)-C(27)	1.382(2)	C(5)-C(4)-C(10)	121.53(12)
C(27)-C(28)	1.383(2)	C(4)-C(3)-C(2)	122.21(12)
C(28)-C(29)	1.4005(18)	C(3)-C(2)-C(1)	119.75(12)
C(29)-C(33)	1.5258(19)	C(3)-C(2)-C(7)	118.96(12)
C(30)-C(31)	1.5363(19)	C(1)-C(2)-C(7)	121.27(11)
C(30)-C(32)	1.537(2)	C(6)-C(5)-C(4)	121.84(12)
C(33)-C(34)	1.531(2)	C(5)-C(6)-C(1)	119.89(12)
C(33)-C(35)	1.5381(19)	C(5)-C(6)-C(13)	118.53(12)
C(36)-C(37)	1.366(9)	C(1)-C(6)-C(13)	121.53(12)
C(36)-C(41)	1.400(7)	C(9)-C(7)-C(2)	110.73(11)
C(36)-C(42)	1.484(7)	C(9)-C(7)-C(8)	109.69(13)
C(37)-C(38)	1.394(9)	C(2)-C(7)-C(8)	112.84(13)
C(38)-C(39)	1.328(8)	C(4)-C(10)-C(11)	112.72(12)
C(39)-C(40)	1.355(7)	C(4)-C(10)-C(12)	110.90(12)
C(40)-C(41)	1.405(8)	C(11)-C(10)-C(12)	110.37(12)
C(43)-C(48)	1.374(6)	C(6)-C(13)-C(14)	113.05(12)
C(43)-C(44)	1.419(8)	C(6)-C(13)-C(15)	109.84(12)
C(43)-C(49)	1.508(7)	C(14)-C(13)-C(15)	109.95(12)
C(44)-C(45)	1.368(8)	N(1)-C(16)-C(17)	109.01(11)
C(45)-C(46)	1.339(9)	N(1)-C(16)-P(1)	117.55(9)
C(46)-C(47)	1.311(9)	C(17)-C(16)-P(1)	132.87(10)
C(47)-C(48)	1.376(7)	C(21)-C(17)-C(20)	109.98(14)

C(21)-C(17)-C(16)	109.54(13)	C(38)-C(39)-C(40)	121.8(7)
C(20)-C(17)-C(16)	112.86(12)	C(39)-C(40)-C(41)	120.7(6)
C(21)-C(17)-C(18)	110.91(12)	C(36)-C(41)-C(40)	118.2(6)
C(20)-C(17)-C(18)	111.07(13)	C(48)-C(43)-C(44)	118.8(5)
C(16)-C(17)-C(18)	102.28(10)	C(48)-C(43)-C(49)	120.7(6)
C(19)-C(18)-C(17)	107.13(11)	C(44)-C(43)-C(49)	120.5(6)
N(1)-C(19)-C(23)	112.82(10)	C(45)-C(44)-C(43)	117.1(6)
N(1)-C(19)-C(18)	99.66(10)	C(46)-C(45)-C(44)	122.8(7)
C(23)-C(19)-C(18)	111.89(11)	C(47)-C(46)-C(45)	120.0(7)
N(1)-C(19)-C(22)	109.73(11)	C(46)-C(47)-C(48)	121.7(7)
C(23)-C(19)-C(22)	108.35(11)	C(43)-C(48)-C(47)	119.5(6)
C(18)-C(19)-C(22)	114.27(11)		
C(29)-C(24)-C(25)	122.03(11)		
C(29)-C(24)-N(1)	120.39(11)		
C(25)-C(24)-N(1)	117.56(11)		
C(26)-C(25)-C(24)	117.39(13)		
C(26)-C(25)-C(30)	118.86(12)		
C(24)-C(25)-C(30)	123.60(12)		
C(27)-C(26)-C(25)	121.69(13)		
C(26)-C(27)-C(28)	119.57(12)		
C(27)-C(28)-C(29)	121.74(13)		
C(28)-C(29)-C(24)	117.38(12)		
C(28)-C(29)-C(33)	118.25(12)		
C(24)-C(29)-C(33)	124.25(11)		
C(25)-C(30)-C(31)	112.71(12)		
C(25)-C(30)-C(32)	110.29(12)		
C(31)-C(30)-C(32)	109.32(12)		
C(29)-C(33)-C(34)	112.51(12)		
C(29)-C(33)-C(35)	110.24(11)		
C(34)-C(33)-C(35)	109.25(12)		
C(37)-C(36)-C(41)	118.4(6)		
C(37)-C(36)-C(42)	123.2(6)		
C(41)-C(36)-C(42)	118.3(6)		
C(36)-C(37)-C(38)	122.1(6)		
C(39)-C(38)-C(37)	118.8(6)		

(S4) Computational Details:

All geometry optimizations were carried out with Gaussian09[10] utilizing the generalized gradient approximation functional of Becke in conjunction with the correlation functional of Perdew (BP86)[11]. For describing dispersion interactions, Grimme's C^6R^{-6} correction terms with a Becke-Johnson (BJ) damping function (D3(BJ))[12] were included in all optimizations. A def2-TZVP basis set with a triple ζ-quality, augmented with one set of polarization function was used for the geometry optimization.[13] All stationary points were characterized on the potential energy hypersurface by vibrational analysis as minima (all real frequencies). Single point calculations were performed on the optimized geometries using the meta-GGA exchange functional M06-2X[14] with basis set having triple ζ-quality, augmented with two sets of polarization functions (def2-TZVPP).13 Natural Bond Orbital (NBO version 3.1)[15] analyses were performed at the M06-2X/def2-TZVPP//BP86- GD3BJ/def2-TZVP level of theory. The reaction energy (ΔE) was calculated by adding electronic energy at the M06-2X/def2-TZVPP level of theory with zero-point correction calculated at the BP86-GD3BJ/def2-TZVP level of theory.

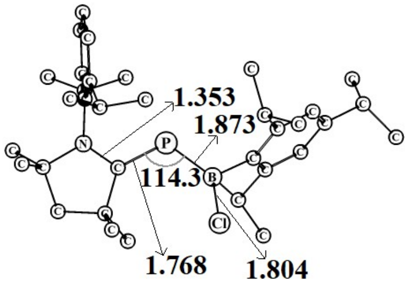

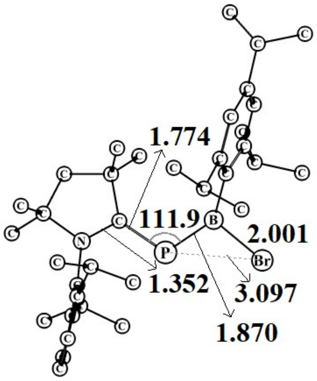

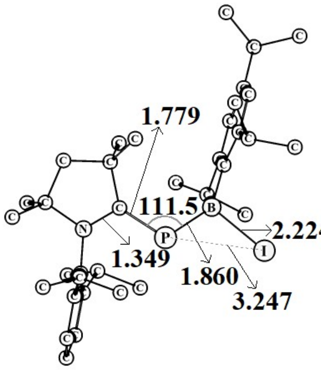

1 **2** **3**

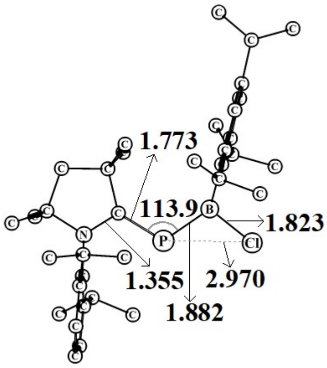

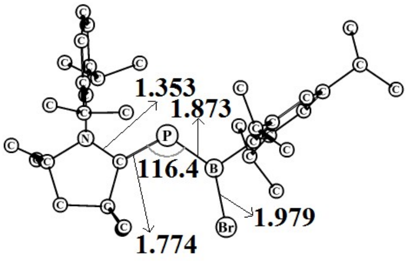

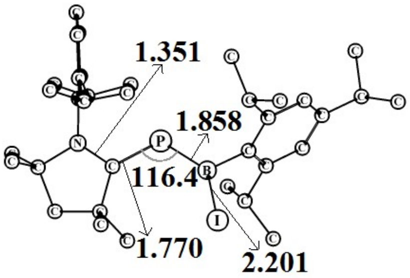

1’ (1.82kcal/mol) **2’** (3.67kcal/mol) **3’**(1.97kcal/mol)

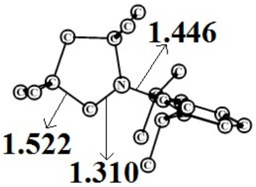

cAAC

Figure S20: Optimized geometries of compounds **1, 2, 3,** their isomers **1’, 2’, 3’,** and cAAC at the BP86/def2-TZVP level of theory using GD3BJ dispersion correction. H atoms attached to C atoms have been omitted for clarity. Relative energy is given in parenthesis.

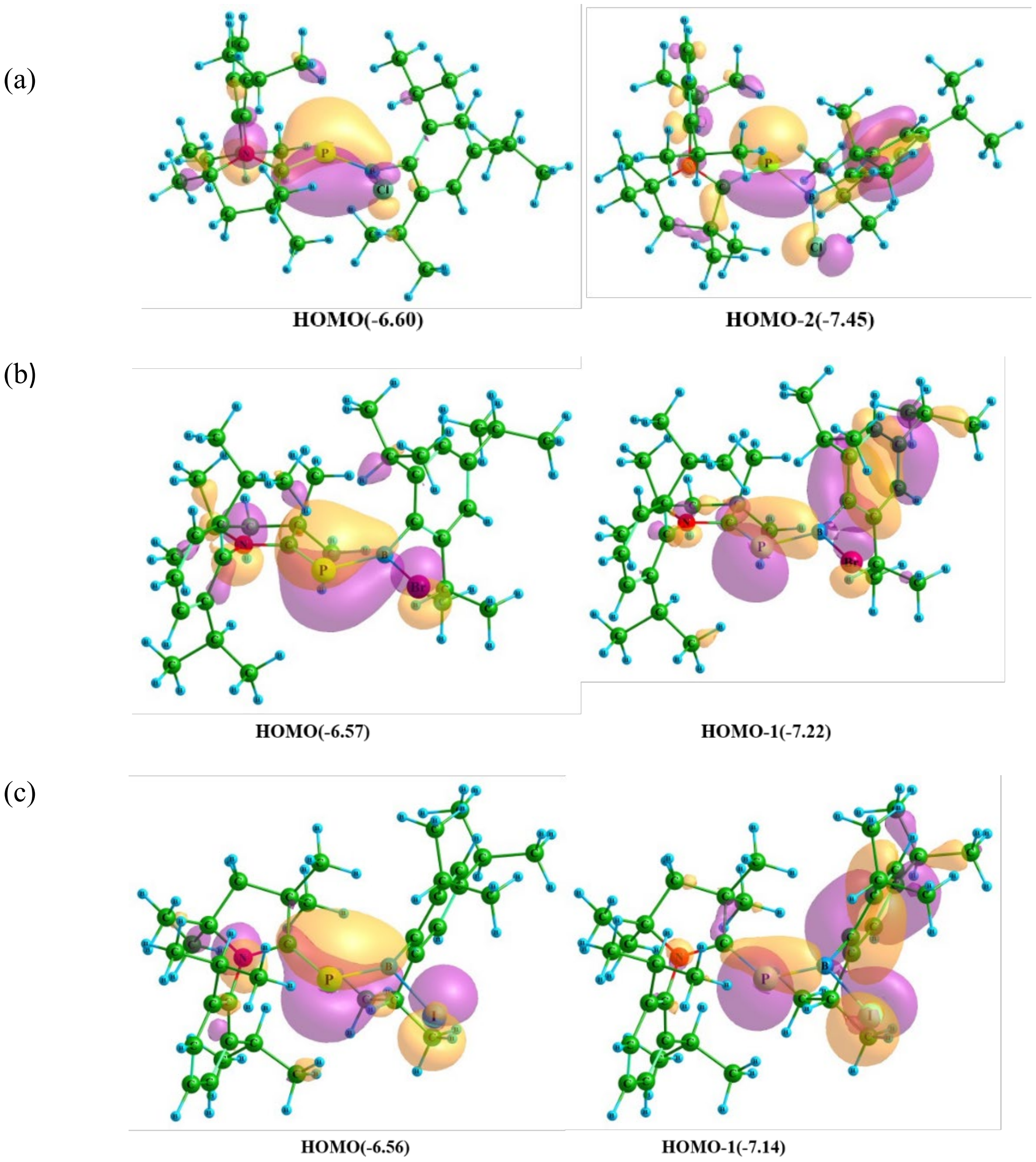

Figure S21: (a) Selected frontier molecular orbitals of (a) **1**, (b) **2** and (c) **3** at the M06-2X/def2-TZVPP//BP86-GD3BJ/def2-TZVP level of theory. Eigen values in eV are given in parenthesis. Isosurface value 0.03

Table S4: Reaction energies (in kcal/mol) for the formation of complexes **1-3** (Scheme 1)

Complex	Energy
**1**	-19.20
**2**	-30.16
**3**	-38.14

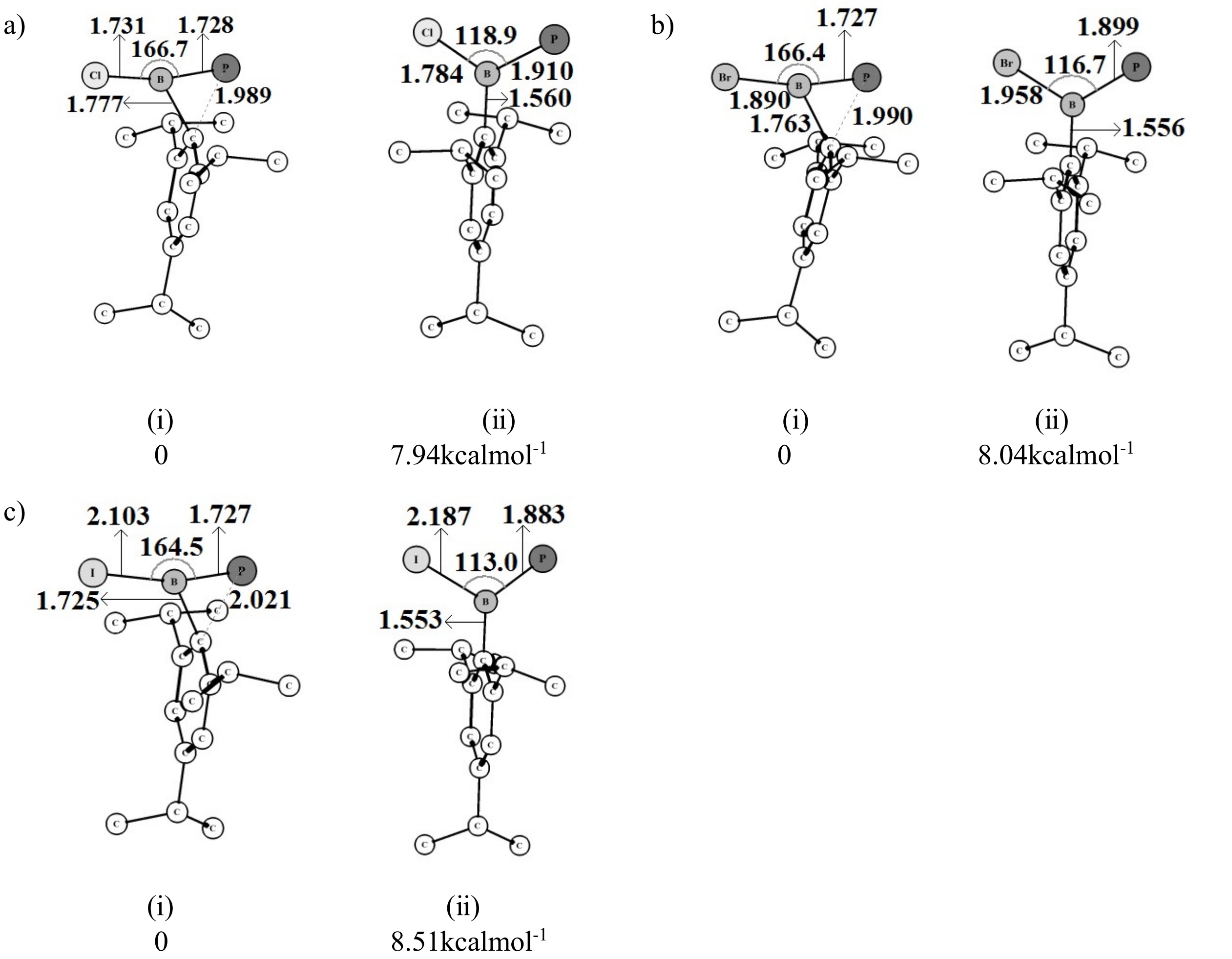

Figure S22: Optimised geometries of singlet (i) and triplet (ii) boryl phosphinidenes PB(Tipp)(X) (X =Cl (a); Br (b); I (c)) at the BP86/def2-TZVP level of theory using GD3BJ dispersion correction; H atoms attached to C atoms have been omitted for clarity; Relative energy of triplet geometry with respect to corresponding singlet geometry is given below the optimized geometry.

Table S5: Reaction energy (ΔE) and Gibbs free energy (ΔG) corresponding to the reaction between singlet and triplet boryl phosphinidenes PB(Tipp)X (X = Cl, Br, I) with singlet cAAC at M062X/def2-TZVPP//BP86/def2-TZVP level of theory

	**Singlet**		**Triplet**	
	**ΔE**	**ΔG**	**ΔE**	**ΔG**
**1**	-70.72	-55.79	-78.66	-60.71
**2**	-71.38	-54.60	-79.41	-60.58
**3**	-70.80	-53.51	-79.31	-60.81

Table S6: Dihedral angles <C-P-B-X, <N-C-B-X (X = Cl, Br, and I) in compounds **1-3** at the BP86-GD3BJ/def2-TZVP level of theory;

	**1**	**2**	**3**
$\theta_{<C\text{-}P\text{-}B\text{-}X}$	23.38	179.07	178.40
$\theta_{<N\text{-}C\text{-}B\text{-}X}$	139.85	29.23	28.66
$\theta_{<N\text{-}C\text{-}P\text{-}B}$	167.76	164.78	162.91

Table S7: Selected second-order interactions and corresponding energies (in kcal/mol) in complexes **1-3** from NBO analyses at the M06-2X/def2-TZVPP//BP86-GD3BJ/def2-TZVP level of theory.

Molecule	Donor Orbital	Acceptor Orbital	Energy
**1**	LP(3)Cl	LP*(1)B	40.74
	LP(2)Cl	BD*(1)P-B	6.89
	LP(1)Cl	BD*(1)P-B	5.14
	LP(1)P	LP*(1)B	5.41
	LP(1)P	BD*(1)C_{cAAC}-C_{adj}	18.27
	LP(1)P	BD*(1)B-Cl	14.92
	BD(2)P-C_{cAAC}	LP*(1)B	38.44
**2**	LP(3)Br	LP*(1)B	35.32
	LP(2)Br	BD*(1)P-B	3.44
	LP(1)Br	BD*(1)P-B	3.16
	LP(1)P	LP*(1)B	2.56
	LP(1)P	BD*(1)C_{cAAC}-C_{adj}	19.03
	LP(1)P	BD*(1)B-C	16.72
	BD(2)P-C_{cAAC}	LP*(1)B	49.64
**3**	LP(3)I	LP*(1)B	30.31
	LP(2)I	BD*(1)P-B	2.88
	LP(1)I	BD*(1)P-B	2.96
	LP(1)P	LP*(1)B	2.72
	LP(1)P	BD*(1)C_{cAAC}-C_{adj}	18.96
	LP(1)P	BD*(1)B-C	17.55
	BD(2)P-C_{cAAC}	LP*(1)B	55.98

Table S8: Natural Charge (q_A), Wiberg Bond Index (WBI), and Bond Occupancy (BO) of selected centres/bonds in complexes **1-3** and cAAC from at the M06-2X/def2-TZVPP//BP86-GD3BJ/def2-TZVP level of theory.

Molecule	Atom	q_A	Bond	WBI	BO
**1**					
	$\mathbf{C_{carbene}}$	0.10	$\mathbf{C_{carbene}}$**-N**	1.29	1.98
	$\mathbf{N_{cAAC}}$	-0.44	$\mathbf{C_{carbene}}$**-P**	1.35	1.96 (33.85%P; 66.15%C)<br>1.77 (65.41%P; 34.59%C)
	**P**	0.07	**P-B**	1.31	1.95
	**B**	0.34	**B-C**	0.85	1.96
	**Cl**	-0.22	**B-Cl**	1.06	1.96
	$\mathbf{cAAC_{group}}$	0.14	**LP(1)P**	-	1.87 (54.22% s; 45.63% p)
**2**					
	$\mathbf{C_{carbene}}$	0.12	$\mathbf{C_{carbene}}$**-N**	1.30	1.98
	$\mathbf{N_{cAAC}}$	-0.44	$\mathbf{C_{carbene}}$**-P**	1.31	1.96 (33.67%P; 66.33%C)<br>1.73 (65.39%P; 34.61%C)
	**P**	0.08	**P-B**	1.36	1.96
	**B**	0.31	**B-C**	0.82	1.96
	**Br**	-0.15	**B-Br**	1.08	1.97
	$\mathbf{cAAC_{group}}$	0.16	**LP(1)P**	-	1.88 (54.41% s; 45.43% p)
**3**					
	$\mathbf{C_{carbene}}$	0.13	$\mathbf{C_{carbene}}$**-N**	1.32	1.98
	$\mathbf{N_{cAAC}}$	-0.43	$\mathbf{C_{carbene}}$**-P**	1.28	1.96 (33.51%P; 66.49%C)<br>1.70 (66.33%P; 33.67%C)
	**P**	0.06	**P-B**	1.41	1.96
	**B**	0.24	**B-C**	0.81	1.96
	**I**	-0.07	**B-I**	1.09	1.95
	$\mathbf{cAAC_{group}}$	0.18	**LP(1)P**	-	1.88 (53.76% s 46.06% p)
**cAAC**	$\mathbf{C_{carbene}}$	0.15	$\mathbf{C_{carbene}}$**-N**	1.52	1.98<br>1.95

**N**	-0.45	**$C_{carbene}$-$C_{adjacent}$**	0.99	1.95
**$C_{adjacent}$**	-0.18			

Table S9: First and second proton affinities (**1PA, 2PA**) of complexes **1-3** at the M06-2X/def2-TZVPP//BP86-GD3BJ/def2-TZVP level of theory. Energies are in kcal/mol.

:

Complex	1PA	2PA
**1**	240.70	141.93
**2**	242.38	137.86
**3**	242.17	137.82

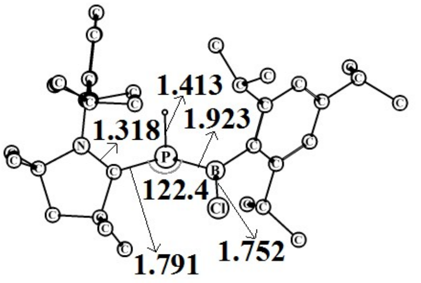

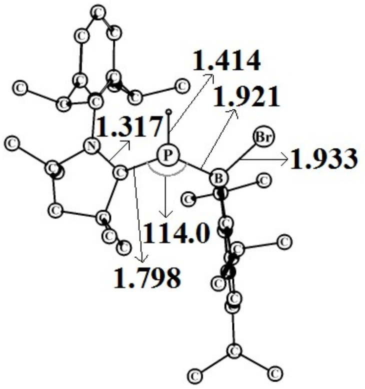

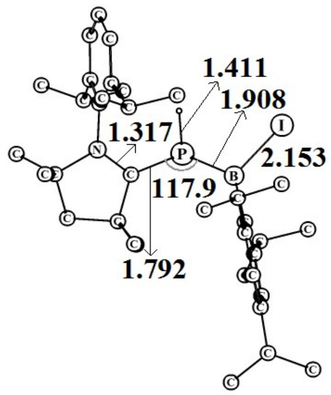

1-H⁺ **2-H⁺** **3-H⁺**

$\theta_P = 34.77^o$ $\theta_P = 41.88^o$ $\theta_P = 35.06\ ^o$

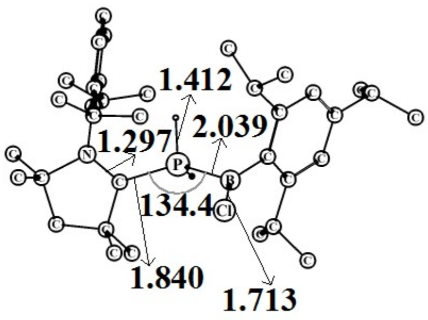

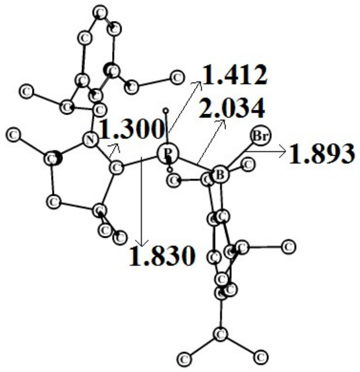

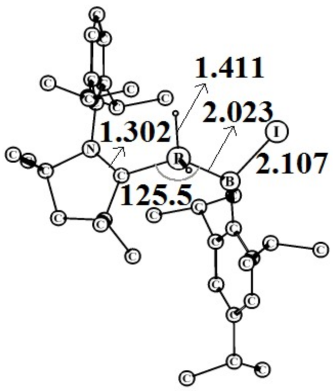

1-2H⁺ **2-2H⁺** **3-2H⁺**

Figure S23: Optimized geometries of singly and doubly protonated complexes at the BP86-GD3BJ/def2-TZVP level of theory. **1-H⁺, 2-H⁺,** and **3-H⁺** are singly protonated adducts, and **1-2H⁺, 2-2H⁺,** and **3-2H⁺** are doubly protonated adducts of **1**, **2,** and **3,** respectively. H atoms attached to C atoms have been omitted for clarity. Pyramidalisation angle (θ_P = 360°-(sum of all angles around the center/atom)) around P atom in complexes **1-H⁺, 2-H⁺, 3-H⁺** are given.

Table S10: Natural Charge (q_A), Wiberg Bond Index (WBI), and Bond Occupancy (BO) of selected centers/bonds in complexes **1H⁺-3H⁺** at the M06-2X/def2-TZVPP//BP86-GD3BJ/def2-TZVP level of theory.

Molecule	Atom	q_A	Bond	WBI	BO
**1-H$^+$**					
	**C$_{carbene}$**	0.20	**C$_{carbene}$-N**	1.51	1.98<br>1.96
	**N$_{cAAC}$**	-0.35	**C$_{carbene}$-P**	1.12	1.96
	**P**	0.33	**P-B**	1.04	1.94
	**B**	0.48	**B-C**	0.88	1.96
	**Cl**	-0.12	**B-Cl**	1.22	1.99
	**H**	0.06	**P-H**	0.94	1.95
	**cAAC$_{group}$**	0.49	**LP(1)P**	-	1.71<br>(37.25% s;<br>62.62% p)
**2-H$^+$**					
	**C$_{carbene}$**	0.22	**C$_{carbene}$-N**	1.52	1.98<br>1.96
	**N$_{cAAC}$**	-0.35	**C$_{carbene}$-P**	1.09	1.96
	**P**	0.31	**P-B**	1.07	1.93
	**B**	0.44	**B-C**	0.85	1.96
	**Br**	-0.04	**B-Br**	1.25	1.97
	**H**	0.06	**P-H**	0.94	1.96
	**cAAC$_{group}$**	0.52	**LP(1)P**	-	1.72<br>(38.19% s;<br>61.65% p)
**3-H$^+$**					
	**C$_{carbene}$**	0.20	**C$_{carbene}$-N**	1.51	1.98<br>1.96
	**N$_{cAAC}$**	-0.35	**C$_{carbene}$-P**	1.10	1.96
	**P**	0.31	**P-B**	1.10	1.94
	**B**	0.36	**B-C**	0.84	1.96
	**I**	0.08	**B-I**	1.28	1.95
	**H**	0.06	**P-H**	0.94	1.96
	**cAAC$_{group}$**	0.51	**LP(1)P**	-	1.68<br>(35.05% s;<br>64.80% p)

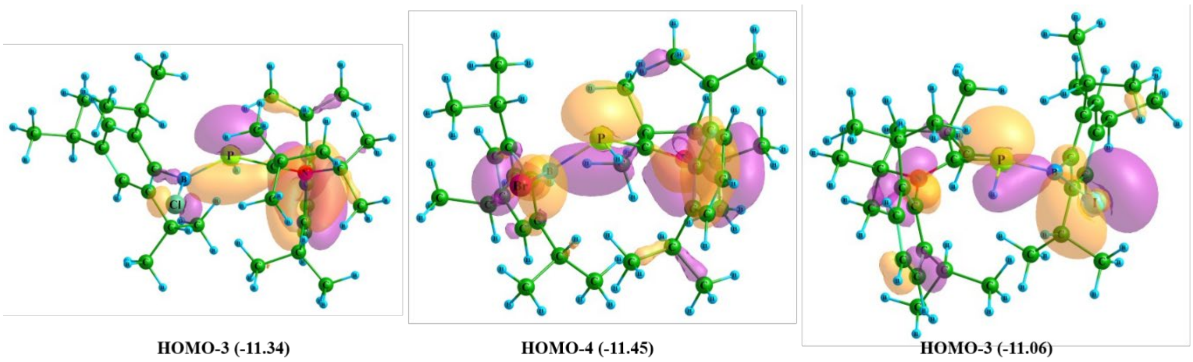

1-H$^+$ **2-H$^+$** **3-H$^+$**

Figure S24: Molecular orbital corresponds to lone pair orbital on P center in **1-H$^+$- 3-H$^+$**) at the M06-2X/def2-TZVPP//BP86-GD3BJ/def2-TZVP level of theory. Eigenvalues in eV are given in parenthesis. Isosurface value 0.03.

Table S11: Optimized Cartesian coordinates, total electronic energy E_{el}(BP86), zero-point energy, ZPE (BP86) at the BP86-GD3BJ/def2-TZVP level of theory and the total electronic energy, E_{el} (M06) at the M06-2X/def2-TZVPP level of theory using Gaussian09 program package. The energies are given in a.u.

1

E_{el}(BP86) = −2248.2526867a.u.
ZPE (BP86) = 0.794711a.u.
E_{el} (M06) = -2247.3480581a.u.

17	-1.231617000	-0.636298000	-3.126951000
6	-2.574426000	-0.023833000	-0.674759000
7	2.894350000	-0.562127000	-0.038942000
15	0.243336000	-0.142178000	-0.154816000
6	-3.058738000	1.297261000	-0.596554000
6	-4.276972000	1.545375000	0.049465000
1	-4.658100000	2.568962000	0.114948000
6	-5.027095000	0.515740000	0.624890000
6	-3.318462000	-1.074119000	-0.092139000
6	-2.253501000	2.445598000	-1.186071000
1	-1.368865000	2.001971000	-1.677287000
6	-4.529564000	-0.791875000	0.545342000
1	-5.100926000	-1.608336000	0.995177000
6	-6.345205000	0.811864000	1.318734000
1	-6.505241000	1.902121000	1.251619000
6	2.076272000	-1.947710000	-1.742064000
6	1.757223000	-0.870713000	-0.704091000
6	4.101087000	-1.384947000	-0.409259000
6	3.441928000	-2.492053000	-1.250901000
1	3.270171000	-3.372408000	-0.612995000
1	4.081921000	-2.806604000	-2.087038000
5	-1.186542000	-0.345251000	-1.346816000
6	2.210383000	-1.284779000	-3.130225000
1	1.262473000	-0.829614000	-3.437738000
1	2.490824000	-2.047764000	-3.872716000
1	2.984573000	-0.506044000	-3.133132000
6	1.036561000	-3.073293000	-1.786509000
1	0.870186000	-3.490289000	-0.783015000
1	1.396534000	-3.879527000	-2.444545000
1	0.077022000	-2.717317000	-2.178184000
6	-1.735455000	3.383727000	-0.084730000
1	-2.571271000	3.840946000	0.467284000
1	-1.128654000	4.194901000	-0.516101000
1	-1.113653000	2.830782000	0.634540000
6	-3.795106000	-3.517181000	-0.636426000
1	-4.650580000	-3.607658000	0.050590000
1	-3.335403000	-4.513553000	-0.724468000
1	-4.183856000	-3.225968000	-1.622798000
6	-2.771718000	-2.494491000	-0.125151000
1	-1.923199000	-2.497112000	-0.833680000
6	-6.297058000	0.435271000	2.809314000
1	-7.236313000	0.709881000	3.314019000
1	-5.466961000	0.945311000	3.319180000
1	-6.152524000	-0.648853000	2.935838000
6	-3.037093000	3.208581000	-2.264349000
1	-3.925700000	3.700834000	-1.839420000
1	-3.375400000	2.527495000	-3.058417000
1	-2.408392000	3.988812000	-2.720394000
6	5.124123000	-0.543872000	-1.186424000
1	4.721968000	-0.150054000	-2.127011000
1	5.996638000	-1.169078000	-1.423452000
1	5.469952000	0.300312000	-0.574007000
6	4.796477000	-1.952617000	0.829669000
1	5.168859000	-1.149172000	1.480486000
1	5.657435000	-2.556431000	0.508347000
1	4.125223000	-2.597541000	1.409286000
6	2.986870000	0.542354000	0.885549000
6	2.819891000	0.313043000	2.270414000
6	3.014145000	1.392004000	3.142701000
1	2.892668000	1.233782000	4.215731000
6	3.330727000	2.660994000	2.665402000
1	3.482420000	3.486693000	3.363170000

6	-7.523175000	0.120039000	0.611952000
1	-8.479069000	0.390432000	1.086828000
1	-7.421743000	-0.975218000	0.660753000
1	-7.569908000	0.404838000	-0.449076000
6	3.411557000	2.882375000	1.292892000
1	3.602512000	3.890035000	0.919587000
6	3.227133000	1.840321000	0.375543000
6	2.329848000	-1.009817000	2.842116000
1	2.263074000	-1.731448000	2.014605000
6	3.267874000	-1.574627000	3.921551000
1	3.284862000	-0.921262000	4.806849000
1	4.301035000	-1.677336000	3.564022000
1	2.916238000	-2.563838000	4.251330000
6	0.908500000	-0.842146000	3.414649000
1	0.228742000	-0.439873000	2.651964000
1	0.911528000	-0.157027000	4.276438000
1	0.519094000	-1.813727000	3.754701000
6	-2.206096000	-2.890355000	1.248361000
1	-2.995119000	-2.866772000	2.016126000
1	-1.412257000	-2.192450000	1.552439000
1	-1.783734000	-3.907426000	1.221587000
6	3.182258000	2.170615000	-1.109200000
1	3.114090000	1.227597000	-1.666974000
6	4.428761000	2.926636000	-1.593979000
1	4.376824000	3.084976000	-2.681659000
1	5.355419000	2.379459000	-1.371105000
1	4.503366000	3.917664000	-1.121562000
6	1.905371000	2.973059000	-1.423202000
1	1.015483000	2.429027000	-1.078996000
1	1.815014000	3.141940000	-2.506862000
1	1.926503000	3.953233000	-0.922774000

2

E_{el}(BP86) = -4362.4245648a.u.
ZPE (BP86) = 0.794796.u.
E_{el} (M06) = -4361.3404842a.u.

35	-1.247790000	-2.211704000	-2.463799000
7	2.492802000	0.423424000	1.034997000
5	-1.012549000	-1.018625000	-0.875105000
15	0.824774000	-0.959397000	-0.533514000
6	-2.333276000	-0.324453000	-0.404035000
6	-3.255741000	-1.012622000	0.423869000
6	-4.415949000	-0.362918000	0.854974000
1	-5.110603000	-0.896543000	1.508292000
6	-3.819992000	1.601439000	-0.386518000
1	-4.054941000	2.618470000	-0.710671000
6	-4.713870000	0.948592000	0.467038000
6	-2.639982000	0.992196000	-0.828365000
6	-3.022046000	-2.473681000	0.787347000
1	-1.956233000	-2.693302000	0.587630000
6	-3.868029000	-3.381925000	-0.124628000
1	-3.660840000	-3.183476000	-1.184130000
1	-4.940241000	-3.205778000	0.055511000
1	-3.655490000	-4.442898000	0.078651000
6	-5.949276000	1.836764000	2.479352000
1	-5.934767000	0.864320000	2.995501000
1	-6.840636000	2.386139000	2.819540000
1	-5.056036000	2.396094000	2.793654000
6	-5.972225000	1.645061000	0.953301000
1	-5.986232000	2.645660000	0.487226000
6	-3.300263000	-2.804780000	2.261544000
1	-3.020755000	-3.847738000	2.473238000
1	-4.369856000	-2.700130000	2.500086000
1	-2.737871000	-2.153565000	2.944227000
6	-1.573285000	3.212787000	-1.539363000
1	-1.268503000	3.414528000	-0.503096000
1	-2.513740000	3.753470000	-1.726171000
1	-0.815498000	3.644885000	-2.210397000
6	2.789199000	1.186535000	2.302421000
6	1.509381000	0.902504000	3.101794000
1	1.224822000	1.751790000	3.738127000
1	1.684259000	0.035778000	3.757529000
6	-1.721182000	1.709553000	-1.807030000
1	-0.723648000	1.247678000	-1.698676000
6	-7.242100000	0.900931000	0.507323000
1	-7.268175000	0.783561000	-0.585590000
1	-8.145443000	1.447031000	0.819896000
1	-7.286805000	-0.104536000	0.953341000
6	0.401120000	0.547558000	2.079723000
6	1.203825000	0.047273000	0.876835000
6	-2.188037000	1.462332000	-3.253291000
1	-1.484794000	1.913284000	-3.970738000
1	-3.181161000	1.909605000	-3.417268000
1	-2.257735000	0.387473000	-3.468058000
6	-0.534568000	-0.520888000	2.649749000
1	0.004131000	-1.459736000	2.841622000
1	-1.356441000	-0.722192000	1.956496000
1	-0.967938000	-0.163430000	3.597015000
6	-0.419219000	1.793716000	1.691072000
1	0.201631000	2.564632000	1.215745000
1	-0.870370000	2.227419000	2.596534000
1	-1.226071000	1.527794000	1.000602000
6	4.034257000	0.658187000	3.015973000
1	4.930555000	0.759616000	2.388121000
1	3.918863000	-0.392938000	3.305050000
1	4.192563000	1.247521000	3.930842000
6	3.007149000	2.673671000	1.992276000
1	3.177516000	3.218873000	2.931491000
1	2.147308000	3.124683000	1.484261000
1	3.895471000	2.804381000	1.359299000
6	3.515637000	0.236435000	0.034437000
6	3.634360000	1.172234000	-1.020230000
6	4.731688000	1.048857000	-1.882308000
1	4.847685000	1.764350000	-2.698415000
6	5.656725000	0.018358000	-1.737170000
1	6.507490000	-0.053944000	-2.417267000
6	5.466511000	-0.945703000	-0.750549000
1	6.155883000	-1.789049000	-0.681924000
6	4.391119000	-0.868153000	0.143487000
6	2.575612000	2.218515000	-1.340428000
1	1.804341000	2.182788000	-0.557877000
6	3.136275000	3.647899000	-1.407978000
1	3.642035000	3.941337000	-0.478269000
1	2.322017000	4.363746000	-1.596280000
1	3.860798000	3.750354000	-2.229827000
6	1.893705000	1.854867000	-2.673862000
1	2.612121000	1.907781000	-3.505910000
1	1.076042000	2.558460000	-2.887666000
1	1.481569000	0.836869000	-2.636508000
6	4.140779000	-2.028025000	1.096482000
1	3.320799000	-1.742375000	1.771216000
6	5.372053000	-2.387586000	1.943159000
1	6.186351000	-2.775292000	1.312633000
1	5.116948000	-3.175562000	2.667526000
1	5.761944000	-1.522597000	2.496980000
6	3.660512000	-3.258496000	0.301458000
1	4.449210000	-3.616021000	-0.378370000
1	2.771729000	-3.012742000	-0.295336000
1	3.405737000	-4.078954000	0.989606000

3

E_{el}(BP86) = - 2085.8339757a.u.
ZPE (BP86) = 0.793968a.u.
E_{el} (M06) = -2084.7696831a.u.

53	1.262531000	3.236213000	0.452047000
6	2.273410000	0.236375000	0.027149000
7	-2.595625000	-1.134002000	-0.493266000
15	-0.866003000	0.897188000	-0.282306000
5	0.971623000	1.093184000	-0.068031000
6	4.609064000	-1.365825000	0.212834000
6	3.735896000	-1.238008000	1.296464000
1	3.969876000	-1.771363000	2.221879000
6	2.579281000	-0.452915000	1.227700000
6	4.316080000	-0.651918000	-0.955153000
1	4.999709000	-0.717427000	-1.805025000
6	3.179812000	0.155269000	-1.060994000
6	1.695110000	-0.289028000	2.455832000
1	0.728174000	0.112288000	2.104221000
6	2.307578000	0.748300000	3.414090000
1	3.268699000	0.384593000	3.810576000
1	1.633624000	0.936570000	4.264183000
1	2.488596000	1.702230000	2.900147000
6	1.412976000	-1.603114000	3.196978000
1	2.331491000	-2.030799000	3.626606000
1	0.967747000	-2.357664000	2.534370000
1	0.718326000	-1.426560000	4.032317000
6	5.847196000	-2.238051000	0.312010000
1	5.846761000	-2.682468000	1.322535000
6	5.809791000	-3.389227000	-0.707390000
1	6.689099000	-4.041789000	-0.593676000
1	5.809404000	-3.001985000	-1.738013000
1	4.904973000	-4.001017000	-0.579093000
6	3.232367000	0.263410000	-3.625089000
1	4.297457000	0.008073000	-3.734207000
1	2.962111000	0.901762000	-4.479519000
1	2.654614000	-0.667887000	-3.694821000
6	2.970930000	1.007221000	-2.306695000
1	1.914962000	1.337300000	-2.304525000
6	7.134688000	-1.409297000	0.164039000
1	7.172172000	-0.600006000	0.907408000
1	7.194125000	-0.949783000	-0.834579000
1	8.025145000	-2.043263000	0.295443000
6	3.855344000	2.266131000	-2.228753000
1	3.663953000	2.828793000	-1.305429000
1	3.662278000	2.930378000	-3.085236000
1	4.919418000	1.982637000	-2.244738000
6	-1.303550000	-0.785048000	-0.662158000
6	-0.549400000	-1.969730000	-1.266262000
6	-1.695444000	-2.899589000	-1.737792000
1	-1.910379000	-2.700021000	-2.798843000
1	-1.426939000	-3.960598000	-1.644040000
6	-2.935886000	-2.546946000	-0.903262000
6	0.323208000	-2.655660000	-0.198215000
1	0.763901000	-3.570978000	-0.621755000
1	-0.259786000	-2.937218000	0.688781000
1	1.138510000	-1.997528000	0.118885000
6	0.328489000	-1.542440000	-2.445004000
1	1.148887000	-0.908514000	-2.096909000
1	-0.253432000	-0.983847000	-3.191864000
1	0.761105000	-2.432501000	-2.927889000
6	-3.114086000	-3.430051000	0.339297000
1	-2.227042000	-3.429694000	0.983354000
1	-3.310273000	-4.464602000	0.023464000
1	-3.974275000	-3.088291000	0.930941000
6	-4.220108000	-2.614585000	-1.729456000
1	-4.420563000	-3.665598000	-1.982732000
1	-4.129125000	-2.051975000	-2.665883000
1	-5.081427000	-2.230605000	-1.165257000
6	-3.588441000	-0.286902000	0.124581000
6	-4.445306000	0.475118000	-0.702441000
6	-5.497286000	1.171076000	-0.093954000
1	-6.171580000	1.764225000	-0.713900000
6	-5.684103000	1.136050000	1.285610000
1	-6.517887000	1.675194000	1.739190000
6	-4.777044000	0.449418000	2.088284000
1	-4.889746000	0.476927000	3.173577000
6	-3.701345000	-0.257257000	1.533986000
6	-4.197035000	0.668044000	-2.191430000
1	-3.406254000	-0.032705000	-2.497037000
6	-5.444586000	0.403619000	-3.049137000
1	-5.192429000	0.474979000	-4.117818000
1	-6.228139000	1.149507000	-2.848714000
1	-5.874412000	-0.589903000	-2.861803000
6	-3.666174000	2.092716000	-2.447178000
1	-2.766847000	2.288194000	-1.847673000
1	-4.427972000	2.842936000	-2.184818000
1	-3.414236000	2.219851000	-3.511080000
6	-2.653054000	-0.840687000	2.470467000
1	-1.909331000	-1.377097000	1.864587000
6	-3.237303000	-1.817466000	3.502876000
1	-2.430264000	-2.244003000	4.117056000
1	-3.782306000	-2.646623000	3.031369000
1	-3.932964000	-1.304435000	4.183581000
6	-1.918788000	0.310255000	3.185858000
1	-1.110561000	-0.086725000	3.817193000
1	-2.609759000	0.871595000	3.832609000
1	-1.483679000	1.006874000	2.455562000

1’

E_{el}(BP86) = -2248.2510937a.u.
ZPE (BP86) = 0.794760a.u.
E_{el} (M06) = -2247.3452127a.u.

17	-1.197259000	-0.665290000	-3.294350000
6	-2.431918000	-0.097226000	-0.794595000
7	2.437929000	-0.151495000	0.983837000
15	0.776816000	-0.371012000	-1.094715000
6	-2.891451000	1.235162000	-0.670126000
6	-4.103906000	1.487575000	-0.018228000
1	-4.458055000	2.516468000	0.088988000
6	-4.881024000	0.455187000	0.515135000
6	-3.238304000	-1.155768000	-0.311593000
6	-2.092149000	2.374882000	-1.284677000
1	-1.065136000	1.997593000	-1.444956000
6	-4.435524000	-0.862002000	0.348557000
1	-5.044949000	-1.681710000	0.736206000
6	-6.175662000	0.758734000	1.247740000
1	-6.311098000	1.854009000	1.220151000
6	0.288240000	-0.272834000	1.915770000
6	1.130389000	-0.236658000	0.637233000
6	2.718524000	-0.329803000	2.452875000
6	1.331308000	-0.039553000	3.042434000
1	1.288758000	1.012481000	3.362002000
1	1.125528000	-0.662440000	3.923750000
5	-1.061051000	-0.367665000	-1.501065000
6	-0.427894000	-1.625830000	2.043504000
1	-1.120835000	-1.758949000	1.206875000
1	-1.003653000	-1.658441000	2.981012000
1	0.278916000	-2.467546000	2.038453000

6	-0.741372000	0.866551000	1.951372000
1	-0.261250000	1.830304000	1.727382000
1	-1.176819000	0.926510000	2.961093000
1	-1.553897000	0.706762000	1.237535000
6	-1.990177000	3.615476000	-0.387965000
1	-2.970421000	4.097102000	-0.250318000
1	-1.322334000	4.360657000	-0.845666000
1	-1.595385000	3.363096000	0.606220000
6	-3.525584000	-3.082908000	-1.887735000
1	-4.620050000	-3.080034000	-1.765268000
1	-3.209397000	-4.108436000	-2.134078000
1	-3.274240000	-2.428989000	-2.732603000
6	-2.847022000	-2.600944000	-0.590488000
1	-1.753751000	-2.614554000	-0.763736000
6	-6.096149000	0.332197000	2.723640000
1	-7.016957000	0.605102000	3.261964000
1	-5.244558000	0.812048000	3.227706000
1	-5.965839000	-0.757750000	2.810167000
6	-2.669044000	2.741230000	-2.664086000
1	-3.698799000	3.118324000	-2.561332000
1	-2.691320000	1.864132000	-3.325170000
1	-2.062223000	3.523966000	-3.145153000
6	3.172689000	-1.766106000	2.750022000
1	2.447483000	-2.504363000	2.383562000
1	3.272663000	-1.892994000	3.837990000
1	4.147947000	-1.977122000	2.295466000
6	3.788210000	0.645085000	2.943311000
1	4.742885000	0.486856000	2.422231000
1	3.953280000	0.483086000	4.018048000
1	3.478449000	1.687165000	2.799386000
6	3.486310000	0.150254000	0.038551000
6	3.739702000	1.515808000	-0.238606000
6	4.799531000	1.827443000	-1.097377000
1	5.012912000	2.873799000	-1.322766000
6	5.576695000	0.826937000	-1.677599000
1	6.403452000	1.090833000	-2.339781000
6	-7.388146000	0.118750000	0.551190000
1	-8.323999000	0.401405000	1.057415000
1	-7.317444000	-0.979702000	0.567127000
1	-7.452996000	0.435188000	-0.499804000
6	5.277748000	-0.509860000	-1.432336000
1	5.860769000	-1.290837000	-1.923851000
6	4.221855000	-0.879073000	-0.586851000
6	2.862296000	2.640590000	0.292088000
1	2.197088000	2.219760000	1.059345000
6	3.673775000	3.774447000	0.938119000
1	4.279657000	4.308859000	0.191502000
1	4.357318000	3.401538000	1.714643000
1	2.997572000	4.511081000	1.397561000
6	1.964578000	3.189100000	-0.833277000
1	1.374268000	2.380529000	-1.286879000
1	2.572199000	3.655763000	-1.623662000
1	1.274030000	3.949169000	-0.437540000
6	-3.159086000	-3.571616000	0.557497000
1	-4.244347000	-3.703595000	0.684926000
1	-2.748316000	-3.224640000	1.515614000
1	-2.735950000	-4.563601000	0.339837000
6	3.866015000	-2.351840000	-0.463875000
1	3.034741000	-2.438332000	0.248853000
6	5.046657000	-3.194354000	0.047625000
1	4.731141000	-4.234778000	0.216764000
1	5.462944000	-2.803944000	0.987528000
1	5.863970000	-3.212009000	-0.689063000
6	3.366778000	-2.900236000	-1.814039000
1	2.523875000	-2.301920000	-2.185314000
1	3.032730000	-3.942694000	-1.699001000
1	4.169045000	-2.883097000	-2.567486000

2'

E_{el}(BP86) = -4362.4190974a.u.
ZPE (BP86) = 0.793658a.u.
E_{el} (M06) = -4361.3335045a.u.

35	1.495490000	-3.191428000	-0.000269000
7	-3.008363000	-0.142587000	-0.393670000
5	1.148862000	-1.244167000	0.056839000
15	-0.363186000	-0.138969000	0.020267000
6	2.466225000	-0.386309000	0.168866000
6	3.184111000	-0.027888000	-0.995146000
6	4.334979000	0.756775000	-0.882616000
1	4.885374000	1.028010000	-1.787597000
6	4.075529000	0.848420000	1.502708000
1	4.429914000	1.197551000	2.477362000
6	4.797254000	1.207441000	0.360798000
6	2.920618000	0.059930000	1.427393000
6	2.716640000	-0.513460000	-2.359520000
1	1.719645000	-0.969002000	-2.215610000
6	3.650279000	-1.606704000	-2.903603000
1	3.717157000	-2.447751000	-2.199098000
1	4.666267000	-1.209836000	-3.056016000
1	3.285380000	-1.990112000	-3.869365000
6	5.887418000	3.397940000	-0.276263000
1	5.752093000	3.230867000	-1.356026000
1	6.779315000	4.029758000	-0.143411000
1	5.011336000	3.953249000	0.088857000
6	6.047269000	2.062657000	0.470211000
1	6.187431000	2.290433000	1.541289000
6	2.546892000	0.635192000	-3.364082000
1	2.125674000	0.263054000	-4.310842000
1	3.512547000	1.110618000	-3.594967000
1	1.875828000	1.408910000	-2.965946000
6	1.600185000	0.918662000	3.406348000
1	0.941289000	1.485688000	2.732706000
1	2.407407000	1.590581000	3.736998000
1	1.019493000	0.626067000	4.294814000
6	-4.231504000	-0.821506000	-0.954858000
6	-3.883757000	-2.293228000	-0.684050000
1	-4.264103000	-2.952380000	-1.476317000
1	-4.351669000	-2.604982000	0.261617000
6	2.167819000	-0.318550000	2.694130000
1	1.306302000	-0.938440000	2.385034000
6	7.293964000	1.304156000	-0.015950000
1	7.427523000	0.364805000	0.539830000
1	8.199989000	1.916389000	0.113117000
1	7.208070000	1.051628000	-1.084176000
6	-2.336638000	-2.384932000	-0.549511000
6	-1.915121000	-0.934449000	-0.307145000
6	3.032848000	-1.175291000	3.630916000
1	2.458020000	-1.486479000	4.516941000
1	3.913673000	-0.614268000	3.979715000
1	3.389757000	-2.077718000	3.113935000
6	-1.996441000	-3.240849000	0.684703000
1	-2.439257000	-2.801188000	1.590689000
1	-0.916008000	-3.332641000	0.832592000
1	-2.415857000	-4.250582000	0.551500000
6	-1.680648000	-2.954341000	-1.818817000
1	-1.955617000	-2.367871000	-2.706722000
1	-2.013652000	-3.991790000	-1.974012000
1	-0.588071000	-2.954567000	-1.729846000
6	-5.507465000	-0.369416000	-0.247085000
1	-5.668021000	0.711695000	-0.362179000

1	-5.482818000	-0.607886000	0.822936000
1	-6.365782000	-0.892179000	-0.692632000
6	-4.359575000	-0.536233000	-2.458525000
1	-5.191065000	-1.129539000	-2.866019000
1	-3.444613000	-0.809074000	-3.000410000
1	-4.575760000	0.522423000	-2.645975000
6	-3.029440000	1.223583000	0.071094000
6	-2.755917000	2.295448000	-0.803421000
6	-2.851856000	3.597940000	-0.293277000
1	-2.645033000	4.441979000	-0.953796000
6	-3.175983000	3.831309000	1.039682000
1	-3.242600000	4.853859000	1.416017000
6	-3.387449000	2.755791000	1.900154000
1	-3.604879000	2.943098000	2.952978000
6	-3.314530000	1.435709000	1.441628000
6	-2.276623000	2.101944000	-2.232655000
1	-2.263078000	1.022762000	-2.438713000
6	-3.201367000	2.790178000	-3.250629000
1	-4.251876000	2.488997000	-3.128712000
1	-2.890375000	2.548415000	-4.277913000
1	-3.160428000	3.884411000	-3.140170000
6	-0.833234000	2.614520000	-2.390478000
1	-0.781291000	3.703844000	-2.241104000
1	-0.462168000	2.393886000	-3.402644000
1	-0.171319000	2.129331000	-1.660085000
6	-3.465479000	0.293932000	2.436616000
1	-3.562976000	-0.641965000	1.867667000
6	-4.714577000	0.439328000	3.319845000
1	-4.631626000	1.304517000	3.994230000
1	-4.840738000	-0.454654000	3.948683000
1	-5.626787000	0.572093000	2.720166000
6	-2.197088000	0.167322000	3.301414000
1	-2.046036000	1.074271000	3.906644000
1	-1.307829000	0.025699000	2.671839000
1	-2.282350000	-0.690662000	3.985810000

3’

E_{el}(BP86) = -2085.8312449a.u.
ZPE (BP86) = 0.793222a.u.
E_{el} (M06) = -2084.7657926a.u.

53	1.433670000	-3.023484000	0.657921000
6	2.418362000	-0.116389000	-0.081841000
7	-3.089836000	-0.060566000	-0.454064000
15	-0.427761000	0.097925000	-0.226192000
5	1.089217000	-0.950675000	0.004284000
6	4.775218000	1.427812000	-0.323202000
6	4.253417000	0.738451000	-1.422748000
1	4.776085000	0.804175000	-2.381838000
6	3.086762000	-0.027532000	-1.320781000
6	4.096528000	1.336054000	0.899251000
1	4.487351000	1.872766000	1.768041000
6	2.930604000	0.577104000	1.037050000
6	2.528269000	-0.759830000	-2.531486000
1	1.645040000	-1.326590000	-2.181042000
6	3.527250000	-1.776698000	-3.100378000
1	4.433521000	-1.276284000	-3.475340000
1	3.081080000	-2.336590000	-3.936758000
1	3.833002000	-2.494479000	-2.325504000
6	2.028899000	0.223899000	-3.600398000
1	2.852225000	0.852482000	-3.973874000
1	1.258007000	0.888275000	-3.183121000
1	1.597477000	-0.315618000	-4.458171000
6	6.043640000	2.252521000	-0.455475000
1	6.380077000	2.158589000	-1.502833000
6	5.780611000	3.742537000	-0.179267000
1	6.696046000	4.335910000	-0.328456000
1	5.445497000	3.896592000	0.858161000
1	4.999197000	4.136608000	-0.845128000
6	1.560681000	1.873860000	2.708651000
1	2.325493000	2.662043000	2.788532000
1	1.020549000	1.824066000	3.666755000
1	0.847795000	2.169153000	1.924795000
6	2.204370000	0.519182000	2.372843000
1	1.384952000	-0.213945000	2.264456000
6	7.165803000	1.712770000	0.447377000
1	7.365957000	0.652085000	0.237883000
1	6.890508000	1.798064000	1.509893000
1	8.097377000	2.279652000	0.295275000
6	3.112523000	0.020006000	3.505915000
1	3.537415000	-0.964356000	3.261845000
1	2.545473000	-0.070255000	4.445169000
1	3.947091000	0.715421000	3.684123000
6	-1.939877000	-0.763732000	-0.547677000
6	-2.258530000	-2.155869000	-1.091156000
6	-3.672408000	-1.959627000	-1.696999000
1	-3.575735000	-1.721194000	-2.767214000
1	-4.283335000	-2.868616000	-1.609819000
6	-4.318267000	-0.764260000	-0.973213000
6	-2.293783000	-3.161331000	0.079388000
1	-2.568546000	-4.155602000	-0.304960000
1	-3.027909000	-2.875372000	0.844004000
1	-1.310998000	-3.237472000	0.559471000
6	-1.265718000	-2.624224000	-2.160872000
1	-0.276753000	-2.818378000	-1.727616000
1	-1.156610000	-1.868494000	-2.951782000
1	-1.628043000	-3.559227000	-2.615500000
6	-5.246963000	-1.165519000	0.181345000
1	-4.765170000	-1.846497000	0.891628000
1	-6.133172000	-1.670114000	-0.228795000
1	-5.586835000	-0.274692000	0.727512000
6	-5.111662000	0.121772000	-1.935024000
1	-5.989277000	-0.437465000	-2.290036000
1	-4.513328000	0.407743000	-2.808061000
1	-5.469499000	1.031872000	-1.433730000
6	-3.159777000	1.228683000	0.191363000
6	-3.078819000	2.406260000	-0.587058000
6	-3.228487000	3.635553000	0.068118000
1	-3.171007000	4.557119000	-0.513902000
6	-3.414975000	3.703702000	1.446167000
1	-3.529960000	4.672614000	1.935685000
6	-3.412573000	2.534854000	2.203416000
1	-3.501854000	2.596308000	3.289517000
6	-3.273093000	1.278234000	1.601326000
6	-2.718627000	2.407027000	-2.066022000
1	-2.666953000	1.361502000	-2.403481000
6	-3.750894000	3.155288000	-2.925707000
1	-3.488048000	3.076129000	-3.991315000
1	-3.770754000	4.225616000	-2.671187000
1	-4.767719000	2.763064000	-2.791285000
6	-1.318804000	3.020013000	-2.272074000
1	-0.569362000	2.494667000	-1.664827000
1	-1.311752000	4.084717000	-1.992468000
1	-1.026291000	2.946199000	-3.330547000
6	-3.145696000	0.048749000	2.488706000
1	-3.121960000	-0.837112000	1.841444000
6	-4.321387000	-0.101038000	3.466947000
1	-4.226506000	-1.039149000	4.034035000
1	-5.290088000	-0.109903000	2.947886000
1	-4.339542000	0.723096000	4.195562000
6	-1.807634000	0.081835000	3.250958000

1	-1.680071000	-0.839892000	3.838323000
1	-1.770599000	0.937429000	3.942235000
1	-0.964617000	0.165755000	2.551826000

1-H^{+}

E_{el}(BP86) = -2248.6504883a.u.
ZPE (BP86) = 0.804009a.u.
E_{el} (M06) = -2247.7409301a.u.

17	-0.964218000	-2.068573000	-2.560344000
6	-2.464109000	-0.395043000	-0.673254000
7	2.896291000	-0.424077000	0.323660000
15	0.182731000	-0.826608000	0.243615000
6	-2.751421000	0.939869000	-1.023396000
6	-3.934549000	1.522095000	-0.559031000
1	-4.173278000	2.552906000	-0.833838000
6	-4.832948000	0.818095000	0.251497000
6	-3.349609000	-1.119749000	0.159126000
6	-1.775855000	1.724571000	-1.882404000
1	-0.958575000	1.027712000	-2.154623000
6	-4.519213000	-0.502781000	0.604026000
1	-5.209573000	-1.063501000	1.238364000
6	-6.113215000	1.471193000	0.736710000
1	-6.134950000	2.490216000	0.314444000
6	2.413913000	-2.621631000	-0.337217000
6	1.908895000	-1.230673000	-0.008776000
6	4.250579000	-1.138364000	0.464047000
6	3.806036000	-2.611248000	0.344069000
1	3.720102000	-3.048647000	1.349562000
1	4.534781000	-3.207209000	-0.219758000
5	-1.147873000	-1.087641000	-1.120515000
6	2.530323000	-2.737646000	-1.876216000
1	1.545619000	-2.677556000	-2.354198000
1	2.975943000	-3.711896000	-2.122608000
1	3.165331000	-1.950540000	-2.302192000
6	1.505092000	-3.734737000	0.200022000
1	1.348144000	-3.639341000	1.283550000
1	1.967342000	-4.712200000	0.002403000
1	0.524508000	-3.727219000	-0.298134000
6	-1.147735000	2.889558000	-1.102365000
1	-1.920578000	3.603767000	-0.782267000
1	-0.422924000	3.433468000	-1.725143000
1	-0.625170000	2.542343000	-0.197510000
6	-4.068526000	-3.544887000	0.035950000
1	-5.049280000	-3.353717000	0.495945000
1	-3.780010000	-4.578199000	0.278063000
1	-4.186904000	-3.461471000	-1.053553000
6	-3.019251000	-2.550567000	0.555322000
1	-2.060121000	-2.816141000	0.064237000
6	-6.130575000	1.595298000	2.269912000
1	-7.038859000	2.117970000	2.603507000
1	-5.257212000	2.154938000	2.634915000
1	-6.120963000	0.603611000	2.747954000
6	-2.410058000	2.200762000	-3.197451000
1	-3.227298000	2.911745000	-3.005451000
1	-2.825320000	1.356320000	-3.764912000
1	-1.664425000	2.709390000	-3.826407000
6	5.238653000	-0.699663000	-0.620886000
1	4.925250000	-0.986760000	-1.630772000
1	6.201993000	-1.187754000	-0.419288000
1	5.403464000	0.385454000	-0.591796000
6	4.858098000	-0.828188000	1.829848000
1	5.086042000	0.241295000	1.932624000
1	5.801447000	-1.384025000	1.922453000
1	4.200858000	-1.138989000	2.649694000
6	2.717412000	1.003302000	0.520891000
6	2.353639000	1.498263000	1.794724000
6	2.180232000	2.884210000	1.916164000
1	1.903318000	3.298659000	2.886404000
6	2.338923000	3.736405000	0.827325000
1	2.199662000	4.811287000	0.951392000
6	-7.354210000	0.721203000	0.222823000
1	-8.272760000	1.243389000	0.527769000
1	-7.398809000	-0.299510000	0.632486000
1	-7.348546000	0.644934000	-0.873830000
6	2.651979000	3.214315000	-0.425037000
1	2.742913000	3.885027000	-1.280030000
6	2.839251000	1.839641000	-0.613553000
6	2.081078000	0.623962000	3.012373000
1	2.228841000	-0.428446000	2.724963000
6	3.039469000	0.952520000	4.172168000
1	2.848152000	1.964115000	4.558676000
1	4.093037000	0.907491000	3.869226000
1	2.887482000	0.247938000	5.002542000
6	0.624319000	0.768478000	3.495266000
1	-0.100792000	0.522344000	2.708491000
1	0.420884000	1.794239000	3.834542000
1	0.443257000	0.094099000	4.344407000
6	-2.797829000	-2.680708000	2.069406000
1	-3.713093000	-2.423747000	2.622672000
1	-1.999978000	-2.003918000	2.410606000
1	-2.519755000	-3.710780000	2.337074000
6	3.082569000	1.310580000	-2.019672000
1	3.377789000	0.258116000	-1.944406000
6	4.212096000	2.059271000	-2.742778000
1	4.431978000	1.574017000	-3.704427000
1	5.136170000	2.077771000	-2.148012000
1	3.933425000	3.100592000	-2.958759000
6	1.785476000	1.340238000	-2.844601000
1	1.009594000	0.717674000	-2.378679000
1	1.966626000	0.954604000	-3.858239000
1	1.393470000	2.363560000	-2.932579000
1	0.214269000	0.584528000	0.310831000

2-H^{+}

E_{el}(BP86) = -4362.8253415a.u.
ZPE (BP86) = 0.804383a.u.
E_{el} (M06) = -4361.7363263a.u.

35	0.841210000	2.727531000	1.991713000
7	-2.256287000	-0.946424000	-0.632345000
5	0.987913000	1.030207000	1.079041000
15	-0.640262000	0.043190000	1.336702000
6	2.287732000	0.530068000	0.420364000
6	3.160419000	-0.358290000	1.095763000
6	4.266785000	-0.863555000	0.409998000
1	4.936848000	-1.557661000	0.919991000
6	3.691719000	0.412092000	-1.550117000
1	3.911987000	0.708104000	-2.578651000
6	4.544146000	-0.499953000	-0.916364000
6	2.567293000	0.931547000	-0.904465000
6	2.924274000	-0.674767000	2.563833000
1	1.837705000	-0.560365000	2.753867000
6	3.650685000	0.362128000	3.443076000
1	3.346504000	1.387298000	3.191350000
1	4.738178000	0.287348000	3.293996000
1	3.436292000	0.187527000	4.507609000
6	5.540508000	-2.602536000	-1.869982000
1	5.504868000	-3.136234000	-0.907784000
1	6.374872000	-3.018075000	-2.453541000

1	4.605124000	-2.812343000	-2.409295000
6	5.731453000	-1.090697000	-1.652342000
1	5.770112000	-0.607685000	-2.643346000
6	3.317303000	-2.099403000	2.973891000
1	2.981339000	-2.305264000	3.999956000
1	4.408994000	-2.232589000	2.959664000
1	2.879400000	-2.857920000	2.309180000
6	1.270376000	1.557839000	-3.023941000
1	0.818319000	0.557414000	-3.082241000
1	2.149807000	1.564060000	-3.683611000
1	0.552276000	2.283136000	-3.433604000
6	-2.445180000	-2.010390000	-1.723763000
6	-1.400355000	-3.039533000	-1.269489000
1	-0.972412000	-3.584339000	-2.119901000
1	-1.880292000	-3.774224000	-0.605804000
6	1.644987000	1.929617000	-1.584191000
1	0.703361000	1.939667000	-0.996660000
6	7.056645000	-0.795901000	-0.929919000
1	7.199241000	0.283880000	-0.781702000
1	7.905612000	-1.178294000	-1.514812000
1	7.087921000	-1.280861000	0.057429000
6	-0.310280000	-2.262802000	-0.480171000
6	-1.078746000	-1.017636000	-0.047286000
6	2.242814000	3.344716000	-1.518061000
1	1.545166000	4.086701000	-1.934333000
1	3.175960000	3.390304000	-2.099356000
1	2.476743000	3.629850000	-0.483428000
6	0.138067000	-3.040753000	0.772490000
1	-0.702566000	-3.238149000	1.452770000
1	0.911528000	-2.489041000	1.324089000
1	0.566773000	-4.004240000	0.461067000
6	0.926781000	-1.972280000	-1.350364000
1	0.690495000	-1.360267000	-2.228260000
1	1.326116000	-2.932579000	-1.705020000
1	1.712380000	-1.467022000	-0.781429000
6	-3.866834000	-2.556990000	-1.742869000
1	-4.601971000	-1.762033000	-1.927731000
1	-4.123524000	-3.072597000	-0.811640000
1	-3.943643000	-3.284210000	-2.563037000
6	-2.123546000	-1.379177000	-3.082488000
1	-2.145753000	-2.164954000	-3.849874000
1	-1.133210000	-0.908459000	-3.100871000
1	-2.873819000	-0.626552000	-3.351891000
6	-3.317989000	-0.035415000	-0.251580000
6	-3.428451000	1.227615000	-0.871508000
6	-4.515486000	2.031728000	-0.499015000
1	-4.634163000	3.011021000	-0.964773000
6	-5.429953000	1.616512000	0.463917000
1	-6.267330000	2.260949000	0.735208000
6	-5.261520000	0.388452000	1.098944000
1	-5.963182000	0.084421000	1.876486000
6	-4.206036000	-0.467561000	0.763114000
6	-2.398415000	1.794097000	-1.836058000
1	-1.604549000	1.045822000	-1.981353000
6	-3.009912000	2.125137000	-3.207693000
1	-3.544177000	1.269940000	-3.643443000
1	-2.224657000	2.434122000	-3.912378000
1	-3.728722000	2.953413000	-3.126509000
6	-1.747516000	3.053469000	-1.233916000
1	-2.487169000	3.856049000	-1.103612000
1	-0.960489000	3.433457000	-1.900425000
1	-1.298818000	2.853649000	-0.251122000
6	-4.042213000	-1.768150000	1.540238000
1	-3.263970000	-2.374324000	1.051866000
6	-5.337685000	-2.598413000	1.566546000
1	-6.116126000	-2.101320000	2.162450000
1	-5.148939000	-3.577948000	2.028455000
1	-5.746070000	-2.762615000	0.559989000
6	-3.569140000	-1.485420000	2.979457000
1	-4.313903000	-0.887003000	3.524144000
1	-2.616982000	-0.936460000	2.997620000
1	-3.427594000	-2.429019000	3.526335000
1	-1.650280000	1.026638000	1.232347000

3-H$^+$

E_{el}(BP86) = -2086.2348609a.u.
ZPE (BP86) = 0.803752a.u.
E_{el} (M06) = -2085.1653946a.u.

53	0.877270000	3.231473000	-0.614284000
6	2.303709000	0.340228000	-0.115591000
7	-2.476567000	-1.315950000	0.137988000
15	-0.677359000	0.379406000	-1.016016000
5	0.995779000	1.088844000	-0.436281000
6	4.625390000	-1.115418000	0.561240000
6	3.845636000	-0.509682000	1.553056000
1	4.148547000	-0.618347000	2.597361000
6	2.693652000	0.215863000	1.238600000
6	4.242160000	-0.953514000	-0.778385000
1	4.858260000	-1.397334000	-1.562312000
6	3.106072000	-0.226833000	-1.139480000
6	1.872059000	0.898828000	2.319656000
1	0.904733000	1.179324000	1.853605000
6	2.558031000	2.197280000	2.774818000
1	3.513176000	1.968144000	3.270829000
1	1.925217000	2.744728000	3.489192000
1	2.772650000	2.857600000	1.923754000
6	1.553554000	0.002083000	3.523306000
1	2.469780000	-0.288482000	4.057297000
1	1.035993000	-0.918449000	3.222329000
1	0.916511000	0.538271000	4.241934000
6	5.855709000	-1.922204000	0.927830000
1	5.965827000	-1.862633000	2.023903000
6	5.676551000	-3.403568000	0.551051000
1	6.551574000	-3.990278000	0.866389000
1	5.567269000	-3.523247000	-0.537800000
1	4.784203000	-3.831673000	1.030749000
6	3.125235000	-1.092411000	-3.558205000
1	4.212028000	-1.237221000	-3.644846000
1	2.749594000	-0.865969000	-4.566099000
1	2.684723000	-2.045495000	-3.233402000
6	2.791045000	0.058117000	-2.600252000
1	1.702339000	0.250975000	-2.673825000
6	7.128397000	-1.336344000	0.293089000
1	7.263736000	-0.281341000	0.570660000
1	7.089004000	-1.395189000	-0.805173000
1	8.014850000	-1.896290000	0.624565000
6	3.513096000	1.347813000	-3.039332000
1	3.261188000	2.193927000	-2.385342000
1	3.240476000	1.613943000	-4.071094000
1	4.602895000	1.203016000	-2.997187000
6	-1.237854000	-1.154689000	-0.279375000
6	-0.466313000	-2.462822000	-0.270459000
6	-1.609277000	-3.495863000	-0.096907000
1	-1.911999000	-3.871786000	-1.085306000
1	-1.288040000	-4.354540000	0.505702000
6	-2.796557000	-2.760244000	0.548124000
6	0.525420000	-2.498071000	0.910409000
1	0.999483000	-3.489225000	0.939500000
1	0.025790000	-2.331688000	1.872766000
1	1.314432000	-1.747600000	0.790559000

6	0.305073000	-2.667553000	-1.582371000
1	1.126140000	-1.943937000	-1.668239000
1	-0.349103000	-2.569488000	-2.460101000
1	0.746981000	-3.674247000	-1.589712000
6	-2.834999000	-2.854388000	2.076504000
1	-1.905172000	-2.516512000	2.548499000
1	-2.999754000	-3.903046000	2.358821000
1	-3.668941000	-2.265478000	2.478870000
6	-4.142317000	-3.213046000	-0.008408000
1	-4.329171000	-4.237951000	0.341050000
1	-4.153141000	-3.224212000	-1.103628000
1	-4.961913000	-2.578917000	0.355771000
6	-3.461056000	-0.256186000	0.230687000
6	-4.315748000	-0.036562000	-0.875105000
6	-5.310172000	0.939574000	-0.734234000
1	-5.987546000	1.131013000	-1.567575000
6	-5.443250000	1.674892000	0.440837000
1	-6.234711000	2.420211000	0.531552000
6	-4.543566000	1.483728000	1.485504000
1	-4.621901000	2.099530000	2.382419000
6	-3.520047000	0.529788000	1.404339000
6	-4.153418000	-0.732248000	-2.220115000
1	-3.395633000	-1.523452000	-2.115730000
6	-5.461315000	-1.380427000	-2.705604000
1	-5.278293000	-1.965434000	-3.618300000
1	-6.212105000	-0.616248000	-2.953238000
1	-5.900786000	-2.045510000	-1.950433000
6	-3.633609000	0.261393000	-3.278073000
1	-2.673526000	0.707685000	-2.982987000
1	-4.353426000	1.078110000	-3.432929000
1	-3.489051000	-0.250379000	-4.240560000
6	-2.482219000	0.482160000	2.517449000
1	-1.761234000	-0.318574000	2.295531000
6	-3.102116000	0.202160000	3.896447000
1	-2.312481000	0.127607000	4.657852000
1	-3.681408000	-0.730285000	3.912211000
1	-3.776092000	1.016894000	4.198292000
6	-1.705350000	1.812563000	2.555117000
1	-0.912299000	1.769554000	3.314580000
1	-2.371276000	2.648009000	2.813503000
1	-1.243481000	2.046160000	1.585960000
1	-1.662429000	1.277670000	-0.554367000

1-2H$^+$

E_{el}(BP86) = -2248.8968824a.u.
ZPE (BP86) = 0.811283a.u.
E_{el} (M06) = -2247.9743845au.

17	-0.890619000	-1.510940000	-3.066720000
6	-2.498110000	-0.275053000	-0.835491000
7	2.852432000	-0.474109000	0.356932000
15	0.126997000	-0.687424000	-0.056152000
6	-2.792453000	1.105884000	-0.935353000
6	-3.909655000	1.590131000	-0.255814000
1	-4.171823000	2.648129000	-0.330006000
6	-4.716705000	0.752821000	0.532753000
6	-3.274246000	-1.134972000	-0.016324000
6	-1.882057000	2.000911000	-1.755060000
1	-1.094340000	1.342051000	-2.185551000
6	-4.375168000	-0.610004000	0.648509000
1	-4.998483000	-1.262731000	1.262718000
6	-5.934133000	1.302176000	1.242262000
1	-5.970768000	2.384313000	1.035440000
6	2.236316000	-2.731117000	0.069487000
6	1.856416000	-1.279122000	0.153549000
6	4.197624000	-1.238993000	0.525083000
6	3.681145000	-2.691088000	0.642545000
1	3.658070000	-2.986550000	1.700771000
1	4.340001000	-3.391047000	0.114451000
5	-1.276335000	-0.875407000	-1.523011000
6	2.182192000	-3.160479000	-1.418087000
1	1.159856000	-3.103091000	-1.817797000
1	2.512519000	-4.206007000	-1.491845000
1	2.834330000	-2.546477000	-2.052364000
6	1.296514000	-3.620642000	0.903064000
1	1.234254000	-3.284646000	1.947952000
1	1.683218000	-4.649014000	0.901912000
1	0.281632000	-3.656979000	0.477892000
6	-1.173315000	3.049980000	-0.884685000
1	-1.901062000	3.740374000	-0.434378000
1	-0.472329000	3.644773000	-1.486476000
1	-0.606045000	2.594735000	-0.055478000
6	-3.848798000	-3.480401000	-0.765166000
1	-4.853131000	-3.448080000	-0.318098000
1	-3.514177000	-4.527174000	-0.772571000
1	-3.933093000	-3.129925000	-1.802985000
6	-2.884804000	-2.601575000	0.046661000
1	-1.887690000	-2.681029000	-0.468054000
6	-5.834678000	1.111488000	2.766425000
1	-6.701976000	1.572812000	3.258551000
1	-4.924570000	1.575552000	3.172935000
1	-5.829437000	0.045241000	3.039554000
6	-2.615640000	2.644152000	-2.941012000
1	-3.396402000	3.332823000	-2.586725000
1	-3.096143000	1.884875000	-3.572802000
1	-1.916451000	3.222525000	-3.561241000
6	5.088014000	-1.001201000	-0.695857000
1	4.645752000	-1.372789000	-1.627906000
1	6.026809000	-1.547429000	-0.529943000
1	5.338528000	0.060634000	-0.810363000
6	4.908921000	-0.751519000	1.781614000
1	5.157228000	0.316250000	1.716827000
1	5.851420000	-1.309126000	1.872097000
1	4.322078000	-0.937436000	2.687834000
6	2.714523000	0.971556000	0.476678000
6	2.376023000	1.506034000	1.743404000
6	2.254030000	2.900341000	1.822915000
1	1.999640000	3.353619000	2.781847000
6	2.450420000	3.714758000	0.710303000
1	2.363242000	4.797682000	0.807024000
6	-7.222027000	0.667946000	0.681064000
1	-8.101359000	1.122267000	1.158300000
1	-7.253437000	-0.413407000	0.882247000
1	-7.305210000	0.817116000	-0.404515000
6	2.738195000	3.147977000	-0.528796000
1	2.859286000	3.792427000	-1.400082000
6	2.866045000	1.761361000	-0.684404000
6	2.090787000	0.673180000	2.986641000
1	2.268061000	-0.388909000	2.753366000
6	3.015178000	1.059760000	4.156217000
1	2.798483000	2.078447000	4.506929000
1	4.075369000	1.024628000	3.875402000
1	2.858276000	0.379531000	5.004955000
6	0.617782000	0.813885000	3.420373000
1	-0.089687000	0.545933000	2.621554000
1	0.393247000	1.847847000	3.717332000
1	0.413258000	0.167458000	4.285246000
6	-2.704259000	-3.111354000	1.480690000
1	-3.660385000	-3.081298000	2.022773000
1	-1.994568000	-2.494887000	2.057157000
1	-2.353550000	-4.152692000	1.489001000

6	3.057254000	1.181170000	-2.077688000
1	3.258092000	0.105581000	-1.984034000
6	4.238994000	1.815135000	-2.827886000
1	4.401576000	1.299714000	-3.784572000
1	5.170169000	1.762206000	-2.247270000
1	4.049361000	2.873674000	-3.054360000
6	1.757555000	1.322621000	-2.887351000
1	0.915105000	0.827611000	-2.378938000
1	1.864455000	0.865916000	-3.881187000
1	1.482485000	2.378200000	-3.022058000
1	0.198913000	0.691291000	0.239562000
1	-0.485668000	-1.266862000	1.075452000

2-2H$^+$

E_{el}(BP86) = -4363.0626673a.u.
ZPE (BP86) = 0.810689a.u.
E_{el} (M06) = -4361.9623283a.u.

35	0.918549000	3.172672000	1.342769000
7	-2.305456000	-1.076375000	-0.355152000
5	1.169831000	1.412141000	0.693532000
15	-0.575385000	0.447269000	1.096436000
6	2.390149000	0.660402000	0.194460000
6	3.292540000	0.038697000	1.093276000
6	4.346610000	-0.699847000	0.559175000
1	5.044174000	-1.198688000	1.233990000
6	3.642823000	-0.176050000	-1.697080000
1	3.805130000	-0.258232000	-2.773938000
6	4.539626000	-0.822479000	-0.829073000
6	2.567957000	0.558044000	-1.203884000
6	3.104213000	0.229984000	2.587760000
1	2.046593000	0.551315000	2.736597000
6	3.998842000	1.375592000	3.094757000
1	3.825343000	2.305814000	2.535959000
1	5.058384000	1.107411000	2.972325000
1	3.817374000	1.568247000	4.161488000
6	5.480081000	-3.138444000	-1.055954000
1	5.492853000	-3.313455000	0.030567000
1	6.287958000	-3.735987000	-1.500510000
1	4.523574000	-3.508683000	-1.453565000
6	5.684367000	-1.645725000	-1.379120000
1	5.670274000	-1.530885000	-2.475527000
6	3.315857000	-1.046393000	3.410743000
1	3.049547000	-0.874350000	4.462713000
1	4.370030000	-1.357610000	3.392408000
1	2.716162000	-1.887490000	3.032497000
6	1.140873000	0.574105000	-3.332546000
1	0.735375000	-0.419594000	-3.094799000
1	1.982003000	0.429955000	-4.025639000
1	0.373931000	1.144487000	-3.874502000
6	-2.516979000	-2.396652000	-1.149673000
6	-1.498571000	-3.303212000	-0.435827000
1	-1.047642000	-4.019886000	-1.132451000
1	-2.007987000	-3.876156000	0.352178000
6	1.596330000	1.324236000	-2.079496000
1	0.686833000	1.495621000	-1.437069000
6	7.045682000	-1.144083000	-0.865834000
1	7.194408000	-0.079341000	-1.093334000
1	7.857704000	-1.711756000	-1.340774000
1	7.140644000	-1.279213000	0.222035000
6	-0.415220000	-2.383868000	0.203765000
6	-1.152351000	-1.066677000	0.245194000
6	2.155976000	2.712751000	-2.428282000
1	1.423596000	3.305709000	-2.994327000
1	3.056198000	2.602344000	-3.050497000
1	2.442105000	3.273950000	-1.528205000
6	-0.084287000	-2.814873000	1.650844000
1	-0.966836000	-2.784243000	2.305980000
1	0.712435000	-2.193304000	2.085005000
1	0.284556000	-3.850006000	1.628083000
6	0.888471000	-2.323863000	-0.615885000
1	0.713631000	-2.020205000	-1.654575000
1	1.330980000	-3.329150000	-0.623547000
1	1.620585000	-1.638378000	-0.172565000
6	-3.955366000	-2.879901000	-1.052196000
1	-4.660664000	-2.136491000	-1.446559000
1	-4.238687000	-3.140936000	-0.027305000
1	-4.047555000	-3.786254000	-1.666568000
6	-2.153584000	-2.109835000	-2.609950000
1	-2.216729000	-3.056446000	-3.164091000
1	-1.135925000	-1.714510000	-2.719606000
1	-2.859432000	-1.407650000	-3.068008000
6	-3.337090000	-0.057538000	-0.227016000
6	-3.415098000	1.002859000	-1.153407000
6	-4.488569000	1.893081000	-1.004356000
1	-4.588320000	2.723883000	-1.703728000
6	-5.418242000	1.747303000	0.020660000
1	-6.247857000	2.449884000	0.108239000
6	-5.278812000	0.717011000	0.948500000
1	-5.995668000	0.632055000	1.765900000
6	-4.239579000	-0.216597000	0.855583000
6	-2.377906000	1.281442000	-2.229708000
1	-1.606141000	0.495538000	-2.193600000
6	-2.993754000	1.278251000	-3.639791000
1	-3.563291000	0.361442000	-3.843538000
1	-2.207956000	1.370759000	-4.402393000
1	-3.681768000	2.125485000	-3.769453000
6	-1.693426000	2.634200000	-1.956484000
1	-2.417575000	3.459186000	-2.001020000
1	-0.919641000	2.834287000	-2.709798000
1	-1.221572000	2.672974000	-0.962164000
6	-4.105136000	-1.286676000	1.933189000
1	-3.360855000	-2.030398000	1.607718000
6	-5.426334000	-2.033838000	2.185773000
1	-6.170595000	-1.380215000	2.660989000
1	-5.256709000	-2.880006000	2.865930000
1	-5.867442000	-2.418944000	1.256673000
6	-3.590035000	-0.666911000	3.247067000
1	-4.296387000	0.083045000	3.630154000
1	-2.619672000	-0.161867000	3.121663000
1	-3.469835000	-1.441735000	4.017243000
1	-1.727131000	1.262438000	1.156082000
1	-0.392253000	0.000628000	2.425076000

3-2H$^+$

E_{el}(BP86) = -2086.4733618a.u.
ZPE (BP86) = 0.810032a.u.
E_{el} (M06) = -2085.3913081a.u.

53	-0.887020000	-3.392197000	-0.155975000
6	-2.405151000	-0.446749000	0.087971000
7	2.352568000	1.289156000	0.054222000
15	0.578881000	-0.620015000	0.865228000
5	-1.160887000	-1.319256000	0.104768000
6	-4.602511000	1.291239000	-0.049418000
6	-4.360977000	0.524812000	1.101508000
1	-5.045584000	0.625848000	1.946913000
6	-3.274624000	-0.349108000	1.196128000
6	-3.725342000	1.163576000	-1.145411000
1	-3.918225000	1.740954000	-2.051702000

6	-2.632633000	0.309125000	-1.085350000
6	-2.997959000	-1.156886000	2.451420000
1	-2.051743000	-1.712394000	2.270418000
6	-4.091501000	-2.206854000	2.698657000
1	-5.058791000	-1.721424000	2.894232000
1	-3.844592000	-2.827293000	3.571571000
1	-4.216611000	-2.864109000	1.826732000
6	-2.774553000	-0.261281000	3.679684000
1	-3.685446000	0.301430000	3.928712000
1	-1.973467000	0.476075000	3.507670000
1	-2.506066000	-0.864535000	4.558253000
6	-5.785133000	2.233275000	-0.115555000
1	-6.304204000	2.172234000	0.854922000
6	-5.324443000	3.688160000	-0.318292000
1	-6.190611000	4.364147000	-0.310653000
1	-4.814033000	3.814406000	-1.285317000
1	-4.636729000	4.006684000	0.478772000
6	-1.146488000	1.332280000	-2.910601000
1	-1.967956000	1.898369000	-3.372826000
1	-0.435998000	1.083169000	-3.711016000
1	-0.645909000	2.001666000	-2.194680000
6	-1.677324000	0.064879000	-2.238024000
1	-0.794148000	-0.465752000	-1.772795000
6	-6.777728000	1.801783000	-1.211586000
1	-7.118570000	0.767980000	-1.061079000
1	-6.324198000	1.870791000	-2.212000000
1	-7.659022000	2.457998000	-1.200232000
6	-2.281869000	-0.914632000	-3.253049000
1	-2.602790000	-1.847882000	-2.769499000
1	-1.565381000	-1.158117000	-4.050185000
1	-3.169017000	-0.462415000	-3.720930000
6	1.219526000	1.084986000	0.661587000
6	0.553282000	2.342457000	1.164248000
6	1.328044000	3.423329000	0.363395000
1	0.724896000	3.725720000	-0.504160000
1	1.505154000	4.317255000	0.973964000
6	2.656406000	2.804513000	-0.119172000
6	0.828065000	2.421768000	2.691534000
1	0.402259000	3.364986000	3.061406000
1	1.898680000	2.410019000	2.929851000
1	0.338070000	1.601984000	3.235748000
6	-0.956918000	2.420654000	0.926627000
1	-1.510334000	1.651558000	1.482633000
1	-1.214226000	2.325247000	-0.133952000
1	-1.312884000	3.399316000	1.276710000
6	3.866565000	3.182592000	0.733426000
1	3.698053000	3.034040000	1.805867000
1	4.068834000	4.249738000	0.569556000
1	4.758586000	2.623764000	0.423943000
6	2.946094000	3.098509000	-1.586204000
1	3.113528000	4.180580000	-1.683178000
1	2.105130000	2.827759000	-2.235976000
1	3.856160000	2.588883000	-1.927416000
6	3.336593000	0.265473000	-0.277405000
6	3.400109000	-0.229224000	-1.599423000
6	4.451226000	-1.104619000	-1.902409000
1	4.533736000	-1.501968000	-2.914644000
6	5.376992000	-1.490078000	-0.936763000
1	6.194162000	-2.162295000	-1.201260000
6	5.238964000	-1.045438000	0.375326000
1	5.941289000	-1.389578000	1.135157000
6	4.216998000	-0.163232000	0.751024000
6	2.325356000	0.016169000	-2.646733000
1	1.606318000	0.750035000	-2.248803000
6	2.882225000	0.555651000	-3.973777000
1	2.059845000	0.784145000	-4.666416000
1	3.522859000	-0.189890000	-4.465083000
1	3.476432000	1.467569000	-3.837804000
6	1.561568000	-1.298807000	-2.895413000
1	1.148904000	-1.722081000	-1.966670000
1	2.223298000	-2.061512000	-3.328934000
1	0.732863000	-1.138940000	-3.598743000
6	4.088247000	0.211239000	2.222346000
1	3.295739000	0.966306000	2.327460000
6	5.385429000	0.807851000	2.796665000
1	5.220247000	1.148320000	3.828395000
1	5.749211000	1.658533000	2.207137000
1	6.186046000	0.056013000	2.826078000
6	3.668662000	-1.016289000	3.056437000
1	3.518214000	-0.730037000	4.106817000
1	4.444619000	-1.793824000	3.028666000
1	2.738099000	-1.477852000	2.693425000
1	1.708453000	-1.434087000	0.636660000
1	0.370996000	-0.695392000	2.262738000

Me-cAAC=PK

E_{el}(BP86) = -1777.331044a.u.
ZPE (BP86) = 0.454083a.u.
E_{el} (M06) = -1776.7558552a.u.

7	-0.825018000	0.122091000	0.381107000
15	-0.515208000	-0.015864000	-2.426504000
6	-2.869380000	0.214580000	-0.785393000
6	-1.332081000	0.115856000	-0.922339000
6	-1.835486000	0.231904000	1.476940000
6	-3.081723000	0.663964000	0.677309000
1	-3.156950000	1.762440000	0.706067000
1	-4.004315000	0.258391000	1.118756000
6	-3.488422000	-1.167722000	-1.075552000
1	-3.243059000	-1.458086000	-2.107097000
1	-4.584038000	-1.135600000	-0.956527000
1	-3.092301000	-1.946501000	-0.408119000
6	-3.463661000	1.231738000	-1.766292000
1	-3.041712000	2.233033000	-1.592797000
1	-4.559138000	1.284520000	-1.651785000
1	-3.215197000	0.938916000	-2.796479000
6	-2.051141000	-1.102258000	2.213528000
1	-2.369568000	-1.901874000	1.533952000
1	-2.831510000	-0.978177000	2.979673000
1	-1.130231000	-1.419679000	2.722598000
6	-1.447496000	1.282140000	2.526012000
1	-0.493327000	1.028676000	3.012239000
1	-2.222333000	1.323315000	3.306230000
1	-1.362594000	2.281919000	2.082897000
6	0.569789000	-0.014207000	0.636461000
6	1.375774000	1.150607000	0.744923000
6	2.738285000	1.005347000	1.055251000
1	3.361905000	1.896726000	1.157275000
6	3.309677000	-0.257634000	1.230837000
1	4.365733000	-0.351519000	1.494290000
6	2.518784000	-1.399301000	1.076576000
1	2.971632000	-2.386692000	1.194016000
6	1.151571000	-1.303236000	0.767078000
6	0.831942000	2.538522000	0.436160000
1	-0.255921000	2.441626000	0.322883000
6	1.132741000	3.551375000	1.551546000
1	2.212758000	3.748440000	1.635849000
1	0.779943000	3.195394000	2.529433000
1	0.640950000	4.511895000	1.336163000
6	1.369962000	3.039973000	-0.917488000
1	1.036216000	2.362240000	-1.720306000

1	2.471928000	3.104477000	-0.908199000
1	0.978363000	4.044498000	-1.138389000
6	0.369213000	-2.579773000	0.494697000
1	-0.679740000	-2.292802000	0.352962000
6	0.462003000	-3.584248000	1.653758000
1	-0.201376000	-4.442144000	1.468081000
1	0.173738000	-3.130825000	2.612446000
1	1.484842000	-3.977015000	1.763860000
6	0.830490000	-3.229106000	-0.823748000
1	0.645079000	-2.535132000	-1.660154000
1	0.262010000	-4.151541000	-1.016973000
1	1.900404000	-3.496714000	-0.783363000
19	2.532421000	-0.197895000	-2.334803000

di-haloaryl borane (BCl(R))

E_{el}(BP86) = -1531.1956294a.u.
ZPE (BP86) = 0.338441a.u.
E_{el} (M06) = -1530.751339a.u.

17	3.039280000	-0.418378000	-1.511048000
6	0.596442000	-0.019740000	-0.000202000
6	0.100911000	1.299029000	-0.000193000
6	-1.283263000	1.505435000	-0.000104000
1	-1.677128000	2.525630000	-0.000118000
6	-2.185488000	0.436934000	-0.000001000
6	-0.296583000	-1.111856000	-0.000107000
6	1.060842000	2.478014000	-0.000142000
1	2.089092000	2.068800000	-0.000678000
6	-1.672162000	-0.866741000	-0.000005000
1	-2.364022000	-1.712797000	0.000067000
6	-3.683157000	0.687880000	0.000073000
1	-3.825218000	1.782396000	0.000171000
5	2.131968000	-0.269862000	-0.000221000
6	0.916110000	3.327484000	1.272524000
1	-0.084300000	3.783228000	1.329341000
1	1.657916000	4.140315000	1.285077000
1	1.058852000	2.713761000	2.173414000
6	-0.173509000	-3.295417000	-1.271592000
1	-1.267187000	-3.407901000	-1.325406000
1	0.269975000	-4.302498000	-1.283911000
1	0.155394000	-2.760083000	-2.173690000
6	0.237144000	-2.536252000	-0.000052000
1	1.342246000	-2.477164000	-0.000418000
6	-4.348318000	0.127137000	1.268587000
1	-5.423795000	0.360974000	1.280292000
1	-3.891997000	0.548829000	2.175761000
1	-4.242511000	-0.967564000	1.317538000
6	0.915244000	3.328538000	-1.271992000
1	-0.085130000	3.784496000	-1.327716000
1	1.057249000	2.715548000	-2.173496000
1	1.657147000	4.141280000	-1.284411000
6	-4.348361000	0.127364000	-1.268533000
1	-5.423745000	0.361611000	-1.280379000
1	-4.242961000	-0.967383000	-1.317443000
1	-3.891748000	0.548868000	-2.175642000
6	-0.172706000	-3.294950000	1.272028000
1	-1.266360000	-3.407276000	1.326636000
1	0.156891000	-2.759349000	2.173713000
1	0.270675000	-4.302077000	1.284378000
17	3.039069000	-0.418063000	1.510831000

di-haloaryl borane (BBr(R))

E_{el}(BP86) = -5759.5314295a.u.
ZPE (BP86) = 0.337338a.u.
E_{el} (M06) = -5758.7285754a.u.

35	-2.517596000	-0.182479000	1.653408000
5	-1.525002000	-0.084477000	-0.000210000
6	0.018713000	0.065281000	-0.000008000
6	0.836827000	-1.084361000	-0.001518000
6	2.225133000	-0.931735000	-0.001354000
1	2.859150000	-1.822024000	-0.002528000
6	1.994934000	1.462022000	0.001816000
1	2.456498000	2.453432000	0.003120000
6	2.823946000	0.335408000	0.000371000
6	0.600288000	1.348978000	0.001628000
6	0.205783000	-2.467910000	-0.002612000
1	-0.892417000	-2.328146000	-0.004932000
6	0.557201000	-3.251764000	1.271129000
1	0.256121000	-2.695886000	2.170604000
1	1.640963000	-3.434309000	1.333357000
1	0.049467000	-4.228035000	1.279880000
6	4.960493000	-0.117743000	-1.269139000
1	4.785444000	-1.203581000	-1.317499000
1	6.048589000	0.047568000	-1.283024000
1	4.530191000	0.332018000	-2.175549000
6	4.334871000	0.484474000	0.000633000
1	4.552126000	1.566698000	0.002666000
6	0.562244000	-3.252991000	-1.274141000
1	0.053916000	-4.228931000	-1.284184000
1	1.646099000	-3.436487000	-1.331647000
1	0.265386000	-2.697731000	-2.175393000
6	-0.086320000	3.423793000	-1.270459000
1	-0.270187000	2.818669000	-2.169777000
1	0.941174000	3.814195000	-1.330316000
1	-0.774832000	4.282220000	-1.282807000
6	-0.282148000	2.586667000	0.002954000
1	-1.333805000	2.241154000	0.004124000
6	4.960598000	-0.122513000	1.268082000
1	4.530247000	0.323707000	2.176216000
1	6.048668000	0.042901000	1.282555000
1	4.785753000	-1.208563000	1.312295000
6	-0.083446000	3.423551000	1.276066000
1	-0.772281000	4.281684000	1.290306000
1	0.943996000	3.814442000	1.333461000
1	-0.264808000	2.818152000	2.175705000
35	-2.517694000	-0.175741000	-1.654159000

di-haloaryl borane (BI(R))

E_{el}(BP86) = -1206.3461897a.u.
ZPE (BP86) = 0.336669a.u.
E_{el} (M06) = -1205.5842331a.u.

53	2.160037000	-0.120371000	1.847758000
6	-0.469456000	0.092367000	-0.000750000
5	1.073760000	-0.025510000	-0.000651000
6	-3.280156000	0.297914000	0.000071000
6	-2.477105000	1.443636000	0.005900000
1	-2.961835000	2.424128000	0.010738000
6	-1.080430000	1.362935000	0.005608000
6	-2.652672000	-0.955653000	-0.006358000
1	-3.265722000	-1.860678000	-0.010921000
6	-1.261590000	-1.076142000	-0.006752000
6	-0.222878000	2.617651000	0.011890000
1	0.834655000	2.288575000	0.012231000
6	-0.436554000	3.444766000	1.288333000
1	-1.471867000	3.813812000	1.348919000
1	0.234677000	4.316674000	1.305300000
1	-0.241420000	2.839300000	2.185127000

6	-0.432504000	3.454396000	-1.258997000
1	-1.467995000	3.822777000	-1.320737000
1	-0.233269000	2.856039000	-2.159655000
1	0.237899000	4.327074000	-1.266724000
6	-4.793980000	0.412253000	0.001144000
1	-5.035929000	1.489207000	0.006205000
6	-5.406127000	-0.200848000	-1.269999000
1	-6.497575000	-0.059289000	-1.283789000
1	-5.207435000	-1.282515000	-1.320542000
1	-4.985544000	0.260109000	-2.175341000
6	-0.929682000	-3.233957000	-1.284255000
1	-2.009000000	-3.441494000	-1.345367000
1	-0.399141000	-4.197989000	-1.294117000
1	-0.642442000	-2.670795000	-2.183936000
6	-0.594236000	-2.442267000	-0.011806000
1	0.499760000	-2.268902000	-0.017127000
6	-5.404838000	-0.212498000	1.267259000
1	-4.983212000	0.240030000	2.176361000
1	-5.206115000	-1.294594000	1.307540000
1	-6.496262000	-0.071022000	1.283529000
6	-0.917441000	-3.235246000	1.263116000
1	-0.619604000	-2.673708000	2.160392000
1	-0.388596000	-4.200282000	1.266236000
1	-1.996542000	-3.440446000	1.335231000
53	2.162446000	-0.100465000	-1.84856400

References:

[1] S. Kundu, S. Sinhababu, M. M. Siddiqui, A. V. Luebben, B. Dittrich, T. Yang, G. Frenking, H. W. Roesky, *J. Am. Chem. Soc.* **2018**, *140*, 9409–9412.

[2] A. J. Rosenthal, S. Mallet-Ladeira, G. Bouhadir, D. Bourissou, *Synthesis* **2018**, *50*, 3671–3678.

[3] a) D. Stalke, *Chem. Soc. Rev.* **1998**, *27*, 171–178; b) T. Kottke, D. Stalke, *J. Appl. Crystallogr.* **1993**, *26*, 615–619.

[4] Bruker AXS Inc., in *Bruker Apex CCD, SAINT v8.30C* (Ed.: Bruker AXS Inst. Inc.), WI, USA, Madison, **2013**.

[5] L. Krause, R. Herbst-Irmer, D. Stalke, *J. Appl. Crystallogr.* **2015**, *48*, 1907–1913.

[6] L. Krause, R. Herbst-Irmer, G. M. Sheldrick, D. Stalke, *J. Appl. Crystallogr.* **2015**, *48*, 3–10.

[7] G. M. Sheldrick, *Acta Crystallogr.* **2015**, *A71*, 3–8.

[8] G. M. Sheldrick, *Acta Crystallogr.* **2015**, *C71*, 3–8.

[9] C. B. Huebschle, G. M. Sheldrick, B. Dittrich, *J. Appl. Crystallogr.* **2011**, *44*, 1281–1284.

[10] M. J. Frisch, G. W. Trucks, H. B. Schlegel, G. E. Scuseria, M. A. Robb, J. R. Cheeseman, G. Scalmani, V. Barone, B. Mennucci, G. A. Petersson, H. Nakatsuji, M. Caricato, X. Li, H. P. Hratchian, A. F. Izmaylov, J. Bloino, G. Zheng, J. L. Sonnenberg, M. Hada, M. Ehara, K. Toyota, R. Fukuda, J. Hasegawa, M. Ishida, T. Nakajima, Y. Honda, O. Kitao, H. Nakai, T. Vreven, J. A. Montgomery, Jr.,J. E. Peralta, F. Ogliaro, M. Bearpark, J. J. Heyd, E. Brothers, K. N. Kudin, V. N. Staroverov, T. Keith, R. Kobayashi, J. Normand, K. Raghavachari, A. Rendell, J. C. Burant, S. S. Iyengar, J. Tomasi, M. Cossi, N. Rega, J. M. Millam, M. Klene, J. E. Knox, J. B. Cross, V. Bakken, C. Adamo, J. Jaramillo, R. Gomperts, R. E. Stratmann, O. Yazyev, A. J. Austin, R. Cammi, C. Pomelli, J. W. Ochterski, R. L. Martin, K. Morokuma, V. G. Zakrzewski, G. A. Voth, P. Salvador, J. J. Dannenberg, S. Dapprich, A. D. Daniels, O. Farkas, J. B. Foresman, J. V. Ortiz, J. Cioslowskiand, D. J. Fox, Gaussian, Inc., Wallingford CT, **2010**.

[11] a) A. D. Becke, *Phys. Rev. A.* **1988**, *38*, 3098–3100; b) J. P. Perdew, *Phys. Rev. B.***1986**, *33*, 8822–8824.

[12] a) A. D. Grimme, J. Antony, S. Ehrlich, H. Krieg, *J. Phys. Chem.* **2010**, *132*, 154104; b) S. Grimme, S. Ehrlich, L. Goerigk, *J. Comput. Chem*. **2011**, *32,* 1456–1465.

[13] F. Weigend, R. Ahlrichs, *Phys. Chem. Chem. Phys.* **2005**, *7*, 3297–3305.

[14] Y. Zhao, D. G. Truhlar, *Theor. Chem. Acc.* **2008**, *120*, 215–241.

[15] a) A. E. Reed, L. A. Curtissand F. Weinhold, *Chem. Rev.* **1988**, *88*, 899–926; b) NBO 3.1. E. D. Glendening, J. K. Badenhoop, A. E. Reed, J. E. Carpenter, J. A. Bohmann, C. M. Morales, C. R. Landis, F. Weinhold (Theoretical Chemistry Institute, University of Wisconsin, Madison, WI, **2013**).
